# Nr1d1 inhibition mitigates intermittent hypoxia-induced pulmonary hypertension via Dusp1-mediated Erk1/2 deactivation and mitochondrial fission attenuation

**DOI:** 10.1038/s41420-024-02219-5

**Published:** 2024-10-29

**Authors:** Zhou Pan, Yan Yao, Xu Liu, Yixuan Wang, Xinyue Zhang, Shiqian Zha, Ke Hu

**Affiliations:** 1 Department of Respiratory and Critical Care Medicine, Renmin Hospital of Wuhan University, Wuhan, China; 2 Department of Pharmacy, Renmin Hospital of Wuhan University, Wuhan, China

**Keywords:** Mechanisms of disease, RNAi

## Abstract

Intermittent hypoxia (IH) precipitates pulmonary vasoconstriction, culminating in the onset of pulmonary hypertension (PH) among individuals afflicted with sleep apnea. While Nuclear receptor subfamily 1 group D member 1 (Nr1d1) is progressively recognized as pivotal regulator of cellular physiology, the role in the pathogenesis of IH-induced PH remains largely uncharted. The expression of Nr1d1 was examined in IH-induced rodent PH and in IH-treated PASMCs. To elucidate the contribution of Nr1d1 to the development of IH-induced PH, we employed siRNA to modulate Nr1d1 expression in vitro and employed serotype 1 adeno-associated virus (AAV1) in vivo. Nr1d1 levels were elevated in IH-induced rodents PH lung tissues and IH-treated PASMCs. Knocking down Nr1d1 by AAV1 effectively inhibited PH progression in chronic IH-induced PH models. Mechanistic investigations identified dual specificity phosphatase 1 (Dusp1), as a direct target that Nr1d1 trans-repressed, mediating Nr1d1’s regulatory influence on Erk1/2/Drp1 signaling. Nr1d1 deficiency ameliorates mitochondrial dysfunction and fission by restoring Dusp1 dysregulation and Drp1 phosphorylation. Activation of Erk1/2 with PMA reversed the Dusp1-mediated regulation of Drp1 phosphorylation, indicating the involvement of the Erk1/2 pathway in Drp1 phosphorylation controlled by Dusp1. Meanwhile, intermittent hypoxia induced more severe PH in Dusp1 knockout mice compared with wild-type mice. Our data unveil a novel role for Nr1d1 in IH-induced PH pathogenesis and an undisclosed Nr1d1-Dusp1 axis in PASMCs mitochondrial fission regulation.

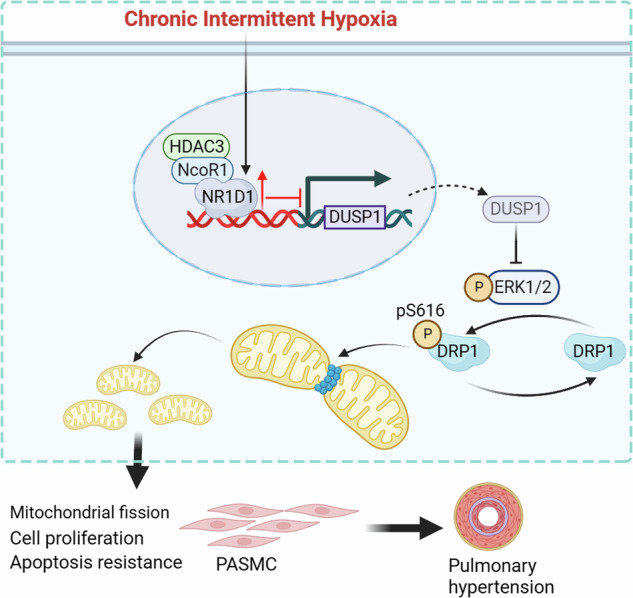

## Introduction

Pulmonary hypertension (PH) represents a severe and often lethal cardiopulmonary disorder characterized by elevated pulmonary arterial pressure, vascular remodeling, and right heart failure [1]. While extensive research has shed light on various contributors to PH, intermittent hypoxia remains a significant etiological factor, especially in cases associated with conditions such as obstructive sleep apnea [2]. A deeper understanding of the intricate molecular mechanisms underlying hypoxia-induced PH is essential to develop targeted therapies for this life-threatening condition.

Mitochondria, beyond the role as ATP generators, assume pivotal functions in oxygen sensing, cell proliferation, apoptosis, and organelle quality control. These noncanonical roles, including the generation of reactive oxygen species (ROS), mitochondrial biogenesis, fission, fusion, and more, intricately govern the behavior of pulmonary circulatory cells in both health and disease [3]. Cell proliferation and resistance to apoptosis in pulmonary artery smooth muscle cells (PASMCs) are major pathophysiologic mechanisms in the development of PH. This enduring “neoplastic phenotype” is sustained in cell culture and arises from shifts in mitochondrial metabolism. These alterations encompass a transition towards uncoupled glycolysis, commonly recognized as the Warburg phenomenon [4], alongside fragmentation of the mitochondrial network [5]. Patients suffering from PH exhibit an abundance of fragmented mitochondria, primarily linked to the increased activation of dynamin-related protein 1 (Drp1), a GTPase known to regulate mitochondrial fission [6]. Han et al. have elucidated that Intermittent hypoxia (IH) elicits concurrent activation of Drp1 and mitochondrial fragmentation, resulting in myocardial injury [7]. Nonetheless, the precise mechanism governing Drp1 activation in IH-induced PH remains to be fully unveiled.

The nuclear receptor subfamily 1 group D member 1 (Nr1d1), known as Rev-Erbα, is recognized for its pivotal role in circadian rhythm regulation and metabolic homeostasis [8]. As part of the nuclear receptor superfamily, it has the capacity to finely regulate gene expression in various pathophysiological contexts and plays a central role in vascular physiology and pathology [9, 10]. Recently, Nr1d1 has emerged as a potential modulator of cardiopulmonary physiology, raising intriguing questions about its involvement in PH pathogenesis [11]. Sun et al. showed that inhibition of nuclear receptor Nr1d1 expression attenuated the development of abdominal aortic aneurysm by targeting the mitochondrial tricarboxylic acid cycle enzyme aconitase-2 [12]. Moreover, Nr1d1 is implicated in the progression of liver fibrosis and the pathogenesis of Parkinson’s disease through its regulatory involvement in Drp1^S616^ phosphorylation [13, 14]. While recent evidence has confirmed the presence of Nr1d1 in cellular physiology, its functional significance in the development of IH-induced PH has remained unexplored.

In this study, our primary aim was to elucidate the potential involvement of Nr1d1 in the pathogenesis of IH-induced PH and uncover the underlying mechanisms. Our investigation reveals that Nr1d1 assumes a pivotal role in IH-induced PH development by directly modulating the activity of the negative regulator of mitogen-activated protein kinase (MAPK) signaling dual specificity phosphatase 1 (Dusp1). Importantly, our findings emphasized that inhibition of extracellular regulating kinase 1/2 (Erk1/2) /Drp1 signaling, a downstream subject to Dusp1 regulation, not only mitigates mitochondrial fission but also effectively restrains the formation of PH.

## Results

### Nr1d1 was up-regulated in IH-treated rodents lung tissue and human primary PASMCs

**Fig. 1:**
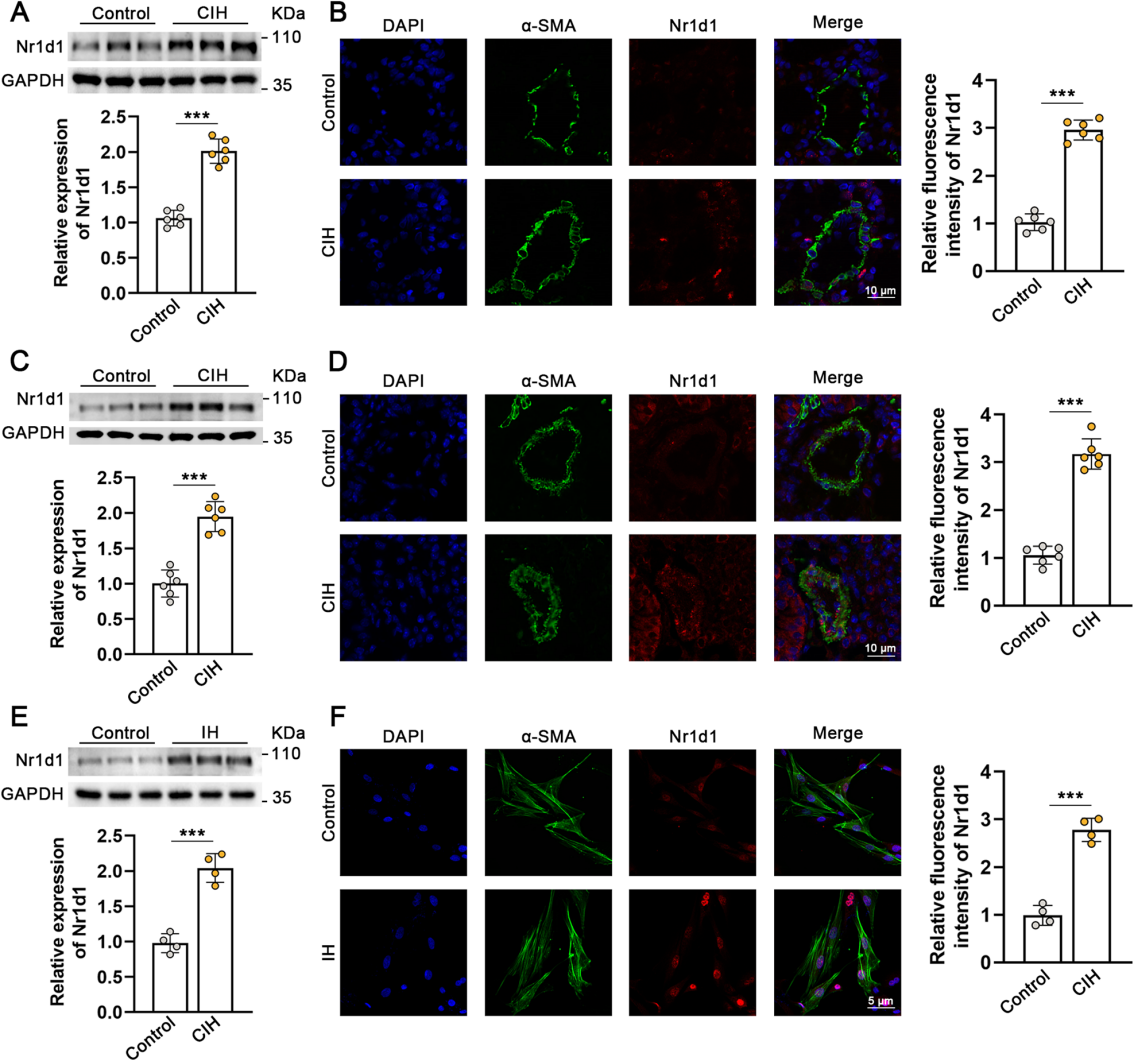
Nr1d1 expression is upregulated in IH-treated rodents and PASMCs. **A** Immunoblot images and the quantitative analysis of Nr1d1 protein expression in rat lung tissues from control group and CIH-induced PH group. **B** Tissue immunofluorescence and semi-quantitative analysis of Nr1d1 in the pulmonary arteries of rats in two groups. Alexa Fluor-488 labels α-SMA as green, Alexa Fluor-594 labels Nr1d1 as red, and DAPI counterstains the nuclei in blue, scale bar = 10 µm. **C** Immunoblot images and the quantitative analysis of Nr1d1 in mouse lung tissue from two groups. **D** Tissue immunofluorescence and semi-quantitative analysis of Nr1d1 in the pulmonary arteries of mouse in two groups, scale bar = 10 µm. **E** Immunoblot images and the quantitative analysis of Nr1d1 protein expression in human PASMCs from control group and IH-treated group. **F** Cellular immunofluorescence double labeling reveals Nr1d1 expression in PASMCs, scale bar = 5 µm. Animal experiments involved 6 biological replicates (*N* = 6) and cell experiments involved 4 biological replicates (*N* = 4). Data are shown as mean ± SD; *P < 0.05 versus Control group.

To invalidate Nr1d1 expression in an IH-induced rodents pulmonary hypertension model, we replicated pulmonary hypertension in rats through 6 weeks of IH exposure and in mice through 8 weeks of IH exposure [15, 16]. The upregulation of Nr1d1 protein was confirmed in rats’ lung tissues (Fig. 1A, B) and murine lung tissues (Fig. 1C, D). At the same time, the expression of α-SMA in lung tissues of the CIH-treated rodents was also increased. Crucially, in double immunofluorescence staining with α-SMA revealed increased Nr1d1 protein presence in pulmonary artery smooth muscle cells in both rat (Fig. 1B) and mouse (Fig. 1D) lung tissues. To further confirm the upregulation of Nr1d1 in PASMCs, human primary PASMCs were treated with IH for 24 h. As shown by western blot, and immunofluorescence staining, Nr1d1 protein levels were significantly increased in the nuclei of IH-treated PASMCs (Fig. 1E, F).

### Inhibiting Nr1d1 effectively improved CIH-induced PH in rats and mice

**Fig. 2:**
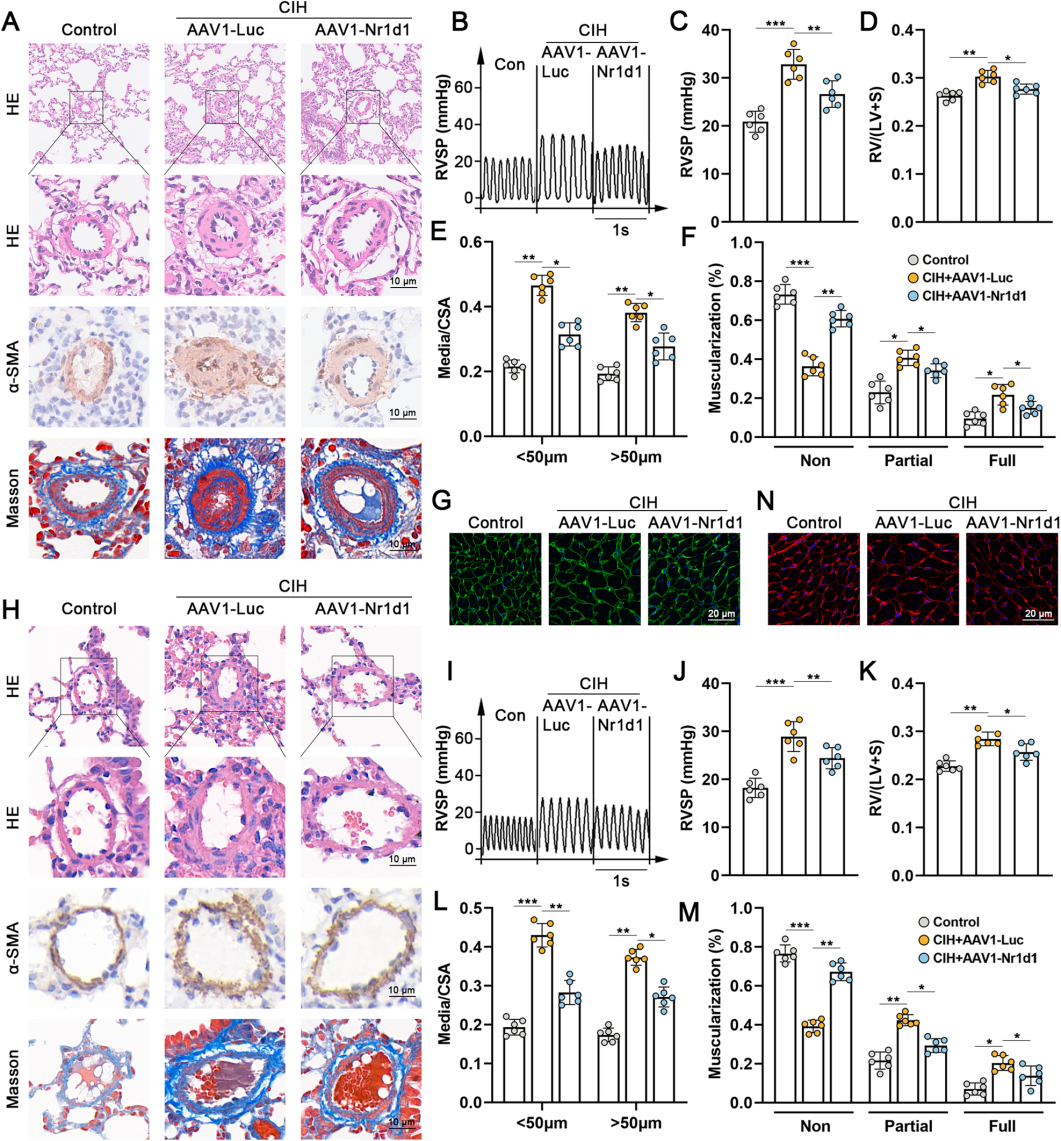
Inhibiting Nr1d1 expression mitigates CIH-induced PH development in rodents. **A** Histological evaluation of rat lung tissues containing peripheral pulmonary vascular in control group, CIH + AAV1-Luc group, and CIH + AAV1-Nr1d1 group through H&E staining, α-SMA immunohistochemistry, and Masson staining, scale bar = 10 µm. **B** Waveforms of right ventricular systolic pressure (RVSP) in three groups of rats. **C** Relative quantitative analysis of RVSP in three groups of rats. **D** Fulton index (right ventricle/ (left ventricle + septum) weight ratio) in three groups of rats. **E** Quantification of vascular medial wall thickness in image A. **F** The ratio of non-muscularized, partially muscularized, and fully muscularized pulmonary arterioles in CIH-induced PH rats. **G** Wheat germ agglutinin (WGA) staining on right ventricular tissues from three rat groups, scale bar = 20 µm. **H** Histological evaluation of mouse lung tissues containing peripheral pulmonary vascular in three mouse groups through H&E staining, α-SMA immunohistochemistry, and Masson staining, scale bar = 10 µm. **I** RVSP in three groups of mice. **J** Relative quantitative analysis of RVSP in three groups of mice. **K** Fulton index in three groups of mice. **L** Quantification of vascular medial wall thickness in image H. **M** The ratio of non-muscularized, partially muscularized, and fully muscularized pulmonary arterioles in CIH-induced PH mice. **N** WGA staining on right ventricular tissues from three mouse groups, scale bar = 20 µm. Involved 6 biological replicates (*N* = 6). Data are shown as mean ± SD; *P < 0.05 verse Control group; ^#^P < 0.05 verse CIH + AAV1-Luc group.

To determine whether the injury in IH-induced PH is mediated by the upregulation of Nr1d1 or if Nr1d1 functions as a self-defensive anti-injury signal, AAV1 was administered via tracheal injection during the 2 weeks of CIH to suppress Nr1d1 expression in lung tissue, with the aim of investigating the influence of Nr1d1 on IH-induced PH (Fig. S1A shown the flowchart of the animal experiment). The findings revealed notable green fluorescent protein expression in the lung tissues of AAV1-Nr1d1-injected rats, particularly in the pulmonary artery wall area (Fig. S1B and D). Western blot results further confirmed a significant suppression of Nr1d1 expression in the lung tissues due to AAV1-Nr1d1 injection (Fig. S1C, E). As expected, the CIH + AAV1-Luc group rats exhibited reduced internal diameters in peripheral pulmonary vessels and increased medial wall thickness in comparison to the normal control group (Fig. 2A, E). This implies extensive remodeling, muscularization of distal pulmonary arteries, and pronounced pulmonary fibrosis around the pulmonary arterial tissues (Fig. 2A, F). Furthermore, a substantial rise in RVSP (Fig. 2B, C), an increased Fulton’s index (Fig. 2D), and hypertrophy of right ventricular cardiomyocytes (Fig. 2D, G) were evident in the CIH + AAV1-Luc group, indicating marked right ventricular hypertrophy. Of greater significance, the inhibition of Nr1d1 expression markedly alleviates IH-induced remodeling of peripheral pulmonary vasculature and right ventricular hypertrophy, among the previously mentioned changes. Similarly, in mice with CIH-induced PH, inhibition of Nr1d1 expression significantly alleviated CIH-induced remodeling and muscularization of peripheral pulmonary arteries, elevated RVSP, and right ventricular hypertrophy, thereby retarding the progression of CIH-induced PH in mice (Fig. 2H–N).

### Suppressing Nr1d1 effectively ameliorated cell proliferation, apoptosis resistance, phenotypic switching, in IH-treated PASMCs

**Fig. 3:**
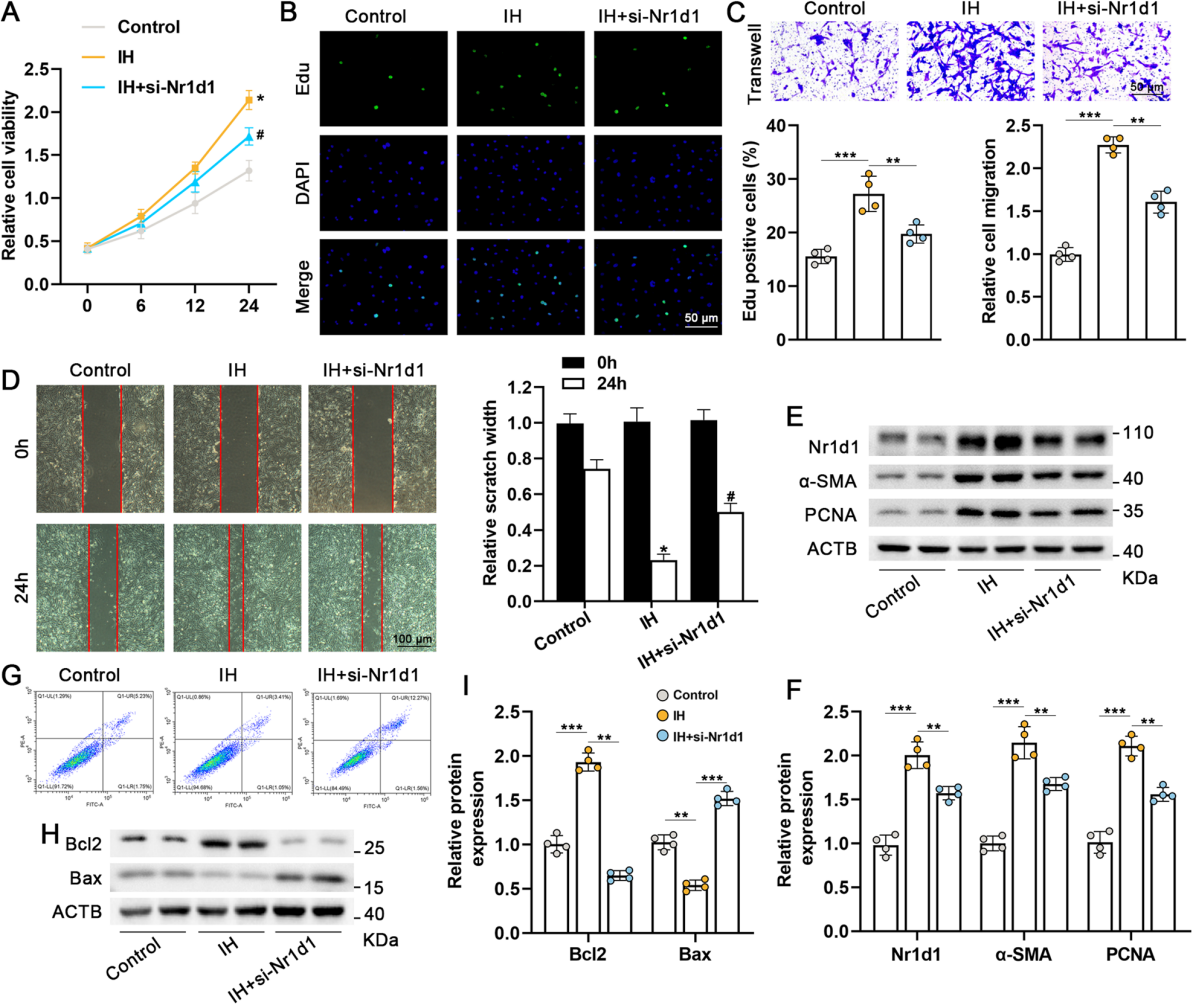
Nr1d1 deficiency prevents proliferation and promotes apoptosis in IH-treated PASMCs. **A** Relative cellular activity of PAMSCs in the three groups (Control, IH, IH+si-Nr1d1 groups) was assessed at 0, 6, 12, and 24 h using the CCK-8 kit. **B** Representative images and quantitative analysis of the proliferation in three group PASMCs using the Edu kit; green color represents positive cells, and DAPI stains the nuclei blue, scale bar = 50 µm. **C** Representative Images and quantitative analysis of the migration in each group of PASMCs reflected by Transwell, scale bar = 50 µm. **D** Representative Images and quantitative analysis of the migration in each group of PASMCs reflected by wound healing experiments, scale bar = 100 µm. **E**, **F** Representative blots and quantitative analysis of Nr1d1, α-SMA and PCNA in the three PASMCs groups. **G** Flow cytometry of apoptosis in the three PASMCs groups. **H**, **I** Representative blots and quantitative analysis of Bcl2 and Bax in the three PASMCs groups. Involved 4 biological replicates (*N* = 4). Data are shown as mean ± SD; *P < 0.05 verse Control group; ^#^P < 0.05 verse IH group.

The neoplastic phenotype of PASMCs is a well-recognized mechanism contributing to the pathogenesis of PH. In this study, employing siRNA to silence Nr1d1 in human primary PASMCs to validate the regulatory effect of Nr1d1 on IH-treated PASMCs. As shown in Fig. S2A, B, both mRNA and protein levels of Nr1d1 were significantly decreased in PASMCs treated with siNr1d1. Deficiency of Nr1d1 markedly impeded cell activity, proliferation and migration ability compared with PASMCS treated IH alone (Fig. 3A–D). Silencing Nr1d1 induced a reduction of α-SMA, PCNA protein expression in PASMCs under intermittent hypoxia conditions (shown in Fig. 3E, F). Moreover, intermittent hypoxia was able to induce an anti-apoptotic phenotype in PASMCs, along with an increase in the anti-apoptotic protein Bcl2 and a decrease in the pro-apoptotic protein Bax (Fig. 3G–I). Whereas Nr1d1 inhibition was able to alleviate IH-induced antiapoptotic phenotype. These findings indicate that Nr1d1 plays a role in the phenotypic transition and survival of PASMCs in the context of IH-induced PH.

### Nr1d1 deficiency prevented IH-induced mitochondrial dysfunction and mitochondrial fission

**Fig. 4:**
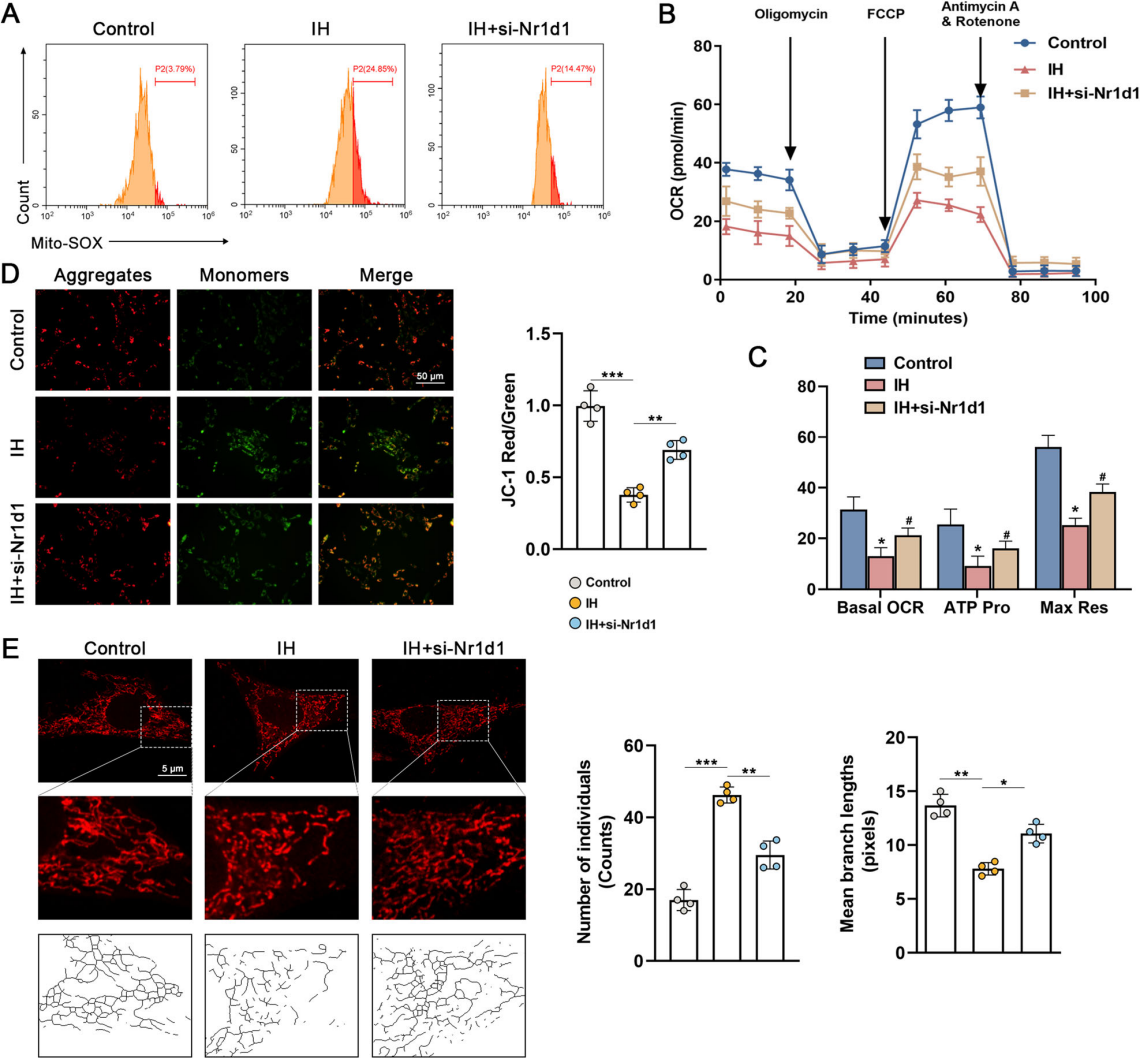
Nr1d1 deficiency mitigates IH-induced mitochondrial dysfunction and fission in PASMCs. **A** Representative flow cytometry images of Mito-SOX staining in three PAMSCs groups. **B** Summarized OCR tracings in PASMCs in indicated groups (*n* = 4 independent experiments). **C** The Basal OCR, maximum respiration, and ATP production in three PASMCs groups. **D** Image and relative quantitative analysis of mitochondrial membrane potential in various groups of PASMCs using the JC-1 kit, scale bar = 50 µm. **E** Representative confocal images of the mitochondrial network of PASMCs examined by MitoTracker Deep Red, and the number of individual mitochondria as well as the mean mitochondrial branch lengths were calculated, scale bar = 5 µm. Involved 4 biological replicates (*N* = 4). Data are shown as mean ± SD; *P < 0.05 verse Control group; ^#^P < 0.05 verse IH group.

Mitochondrial dysfunction and fragmentation are recognized factors in pulmonary hypertension pathogenesis, and prior studies have linked Nr1d1 to their regulation [12, 14]. Thus, we posited that Nr1d1 might influence IH-induced PH by modulating mitochondrial function and fission. As depicted in Fig. 4A, application of the MitoSOX fluorescent dye, a standard approach for assessing mitochondrial superoxide levels, revealed a conspicuous reduction in MitoSOX levels due to Nr1d1 inhibition when contrasted with the IH-exposed PASMCs. Oxygen consumption analysis revealed IH was able to induce mitochondrial aerobic respiration dysfunction in PASMCs (Fig. 4B), while Nr1d1 inhibition significantly increased basal, maximal, and ATP-coupled mitochondrial oxygen consumption rates (Fig. 4B, C). Meanwhile, exposure to intermittent hypoxia also resulted in an increased green-to-red fluorescence ratio post JC-1 staining (Fig. 4D), indicative of mitochondrial membrane potential depolarization when compared to control cells. Significantly, this phenomenon was effectively reversed upon Nr1d1 inhibition. Intermittent hypoxia treatment led to an elevation in mitochondrial fragmentation, characterized by an increase in individual mitochondria and a reduction in mitochondrial branch lengths (Fig. 4E), whereas inhibition of Nr1d1 ameliorated IH-induced mitochondrial fission.

### Nr1d1 directly targeted Dusp1 for transcriptional repression in the regulation of IH-induced PH

**Fig. 5:**
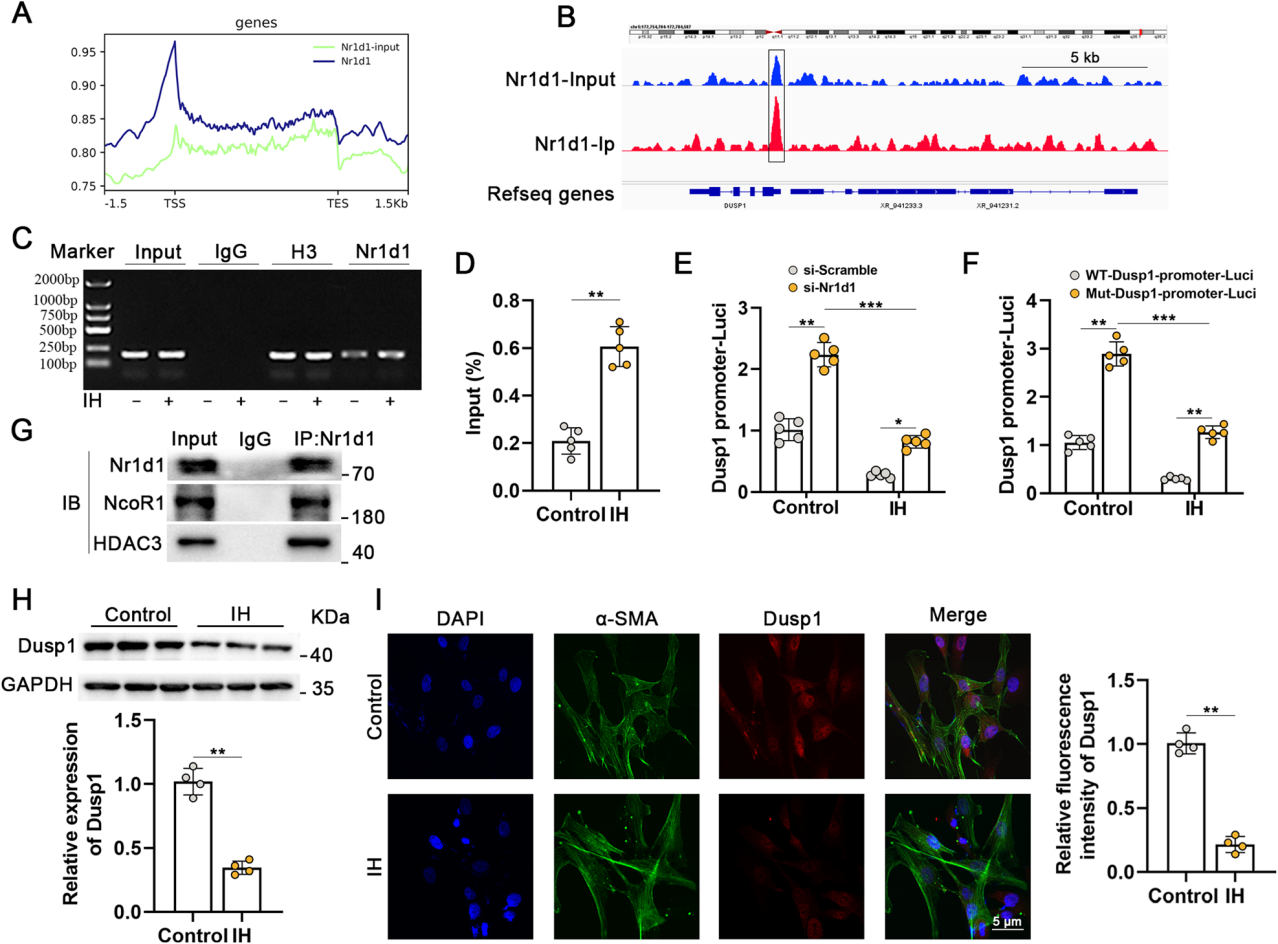
Dusp1 is the direct trans-repression target of Nr1d1 in regulating mitochondrial function. **A** Map of Nr1d1 enrichments relative to the body of RefSeq genes and to 1.5 kbp upstream of the TSS and 1.5 kbp downstream of the TES. X-axis in base-pairs. TSS = Transcription start site, TES = transcription end site. Y-axis average enrichment of CHIP-seq reads over genes and upstream and downstream regions. **B** Nr1d1 profile across the promoter region of Dusp1 on chromosome 5 in human PASMCs of the Nr1d1-Input or Nr1d1-Ip group. **C**, **D** ChIP-qPCR and quantification were conducted using Nr1d1 antibodies to amplify the target promoter region of Dusp1, *n* = 5 biological repetition. **E** After 24 h of normoxia or IH treated, PASMCs were cotransfected with si-Scramble or si-Nr1d1 and pGL3-Dusp1-promoter-Luci plasmid for 48 h. **F** Treated PASMCs were cotransfected with Nr1d1 plasmid and pGL3-m_wtDusp1-promoter-Luci (WT) or pGL3-m_mutDusp1-promoter-Luci plasmid (Mut) for 48 h, *n* = 5 biological repetition. **G** Co-IP of Nr1d1 in PASMCs was performed to confirm the interaction between Nr1d1 with corepressor NCoR1-HDAC3 complex. **H** Immunoblot images and the quantitative analysis of Dusp1 protein expression from control and IH-treated PASMCs. **I** Cellular immunofluorescence double labeling reveals Dusp1 expression in PASMCs. Alexa Fluor-488 labels α-SMA as green, Alexa Fluor-594 labels Dusp1 as red, and DAPI counterstains the nuclei in blue, scale bar = 5 µm. Data are shown as mean ± SD; *P < 0.05, **P < 0.01, ***P < 0.001.

To investigate the downstream mechanisms through which Nr1d1 governs IH-induced PH, we conducted ChIP-seq utilizing antibodies against Nr1d1 in IH-treated human PASMCs. As shown in Fig. 5A, peaks were predominantly intragenic, clustered immediately vicinity to the transcription start site (TSS). It is interesting that enrichment of Nr1d1 at promoter region of Dusp1 on chromosome 5 in human PASMCs of the Nr1d1-Input or Nr1d1-Ip group (Fig. 5B). The binding site of human Nr1d1 in the promoter region of Dusp1 locus (GGATCATGAGGTCAG) was also validated by chromatin immunoprecipitation–polymerase chain reaction. Results showed that IH treatment was able to significantly increase the binding of the Nr1d1 protein to the Dusp1 promoter (Fig. 5C, D). Furthermore, dual luciferase reporter assays revealed that IH treatment reduced Dusp1 promoter activity, and that Nr1d1 inhibition promoted Dusp1 promoter activity (Dusp1-wt, Fig. 5E), whereas the mutant promoter (Dusp1-mut) displayed increased activity compared to the Dusp1-wt promoter in PASMCs (Fig. 5F). Study had shown that Nr1d1 serves as a transcriptional suppressor through the recruitment of NCoR1 (nuclear receptor corepressor 1) and HDAC3 (histone deacetylase 3) [17]. As anticipated, Co-IP findings confirmed the interaction between Nr1d1 and the NCoR1-HDAC3 complex in primary human PASMCs (Fig. 5G). Simultaneously, a significant decrease in the protein expression of Dusp1 was validated in IH-treated PASMCs (Fig. 5H, I). In summary, these investigations have established Dusp1 as the direct trans-repression target of Nr1d1 in the regulation of the IH-induced PH process.

### Silencing Dusp1 reverses the protective effects conferred by Nr1d1 deficiency in IH-treated PASMCs

**Fig. 6:**
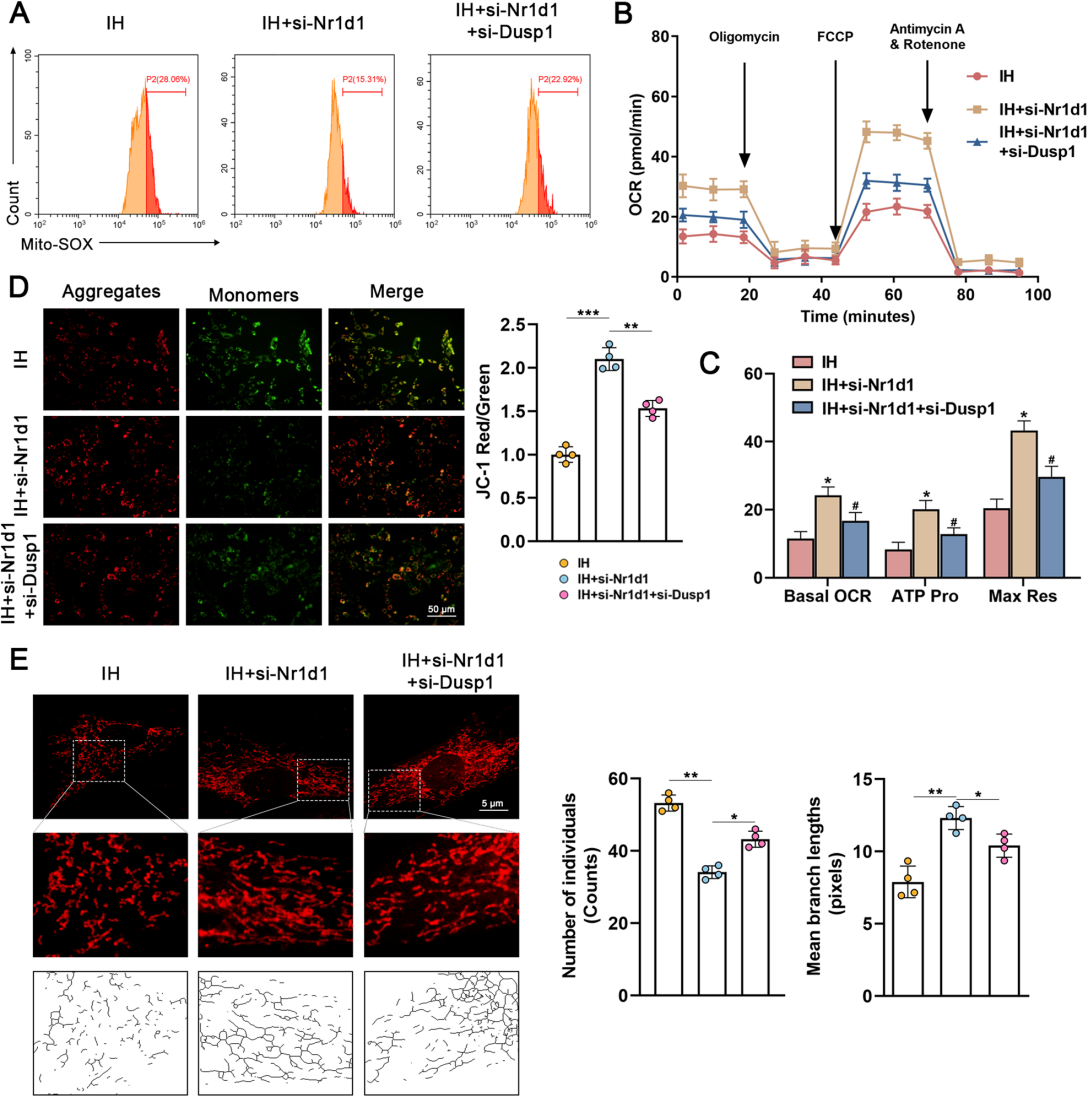
Silencing Dusp1 reverses the protective effects conferred by Nr1d1 deficiency on mitochondrial function. **A** Representative flow cytometry images of Mito-SOX (red) staining in three PAMSCs groups (IH, IH+si-Nr1d1, IH +si-Nr1d1+si-Dusp1). **B** Summarized OCR tracings in PASMCs in indicated groups (*n* = 4 independent experiments). **C** The Basal OCR, maximum respiration, and ATP production in indicated groups of PASMCs. **D** Image and relative quantitative analysis of mitochondrial membrane potential in various groups of PASMCs using the JC-1 kit, scale bar = 50 µm. **E** Representative confocal images of the mitochondrial network of PASMCs examined by MitoTracker Deep Red, and the number of individual mitochondria as well as the mean mitochondrial branch lengths were calculated, scale bar = 5 µm. Involved 4 biological replicates (*N* = 4). Data are shown as mean ± SD; *P < 0.05 verse IH group; ^#^P < 0.05 verse IH+si-Nr1d1 group.

To further confirm that the protective effect of Nr1d1 deficiency on PASMCs is mediated by Dusp1, small interfering RNA (si-Dusp1) targeting Dusp1 mRNA was transfected into PASMCs. As shown in Fig. S3A-D, the deletion of Dusp1 reinstates the decline in cellular activity, proliferation, and migration induced by Nr1d1 inhibition. Importantly, inhibiting Nr1d1 also promotes the expression of Dusp1 protein (Fig. S3E). In intermittent hypoxic conditions, decreased Dusp1 levels restored α-SMA, PCNA, and Bax protein expression, reduced Bax levels, and induced apoptosis resistance in PASMCs, compared to the si-Nr1d1 group (Fig. S3E-I). Similarly, concerning mitochondrial function, the additional silencing of Dusp1 expression, in conjunction with Nr1d1 inhibition, exacerbated the generation of mtROS (Fig. 6A), reducted mitochondrial membrane potential (Fig. 6D), and reduced basal respiration, ATP-coupled, and oxygen consumption rates in PASMCs (Fig. 6B, C). Concurrently, the inhibition of Dusp1 also reversed the protective impact of Nr1d1 deficiency on IH-induced mitochondrial fission (Fig. 6E). Collectively, these findings suggested that Dusp1 mediates the protective effect of Nr1d1 deficiency against IH-induced PH.

### Dusp1 modulates Drp1 phosphorylation and mitochondrial fission via Erk1/2 phosphorylation

**Fig. 7:**
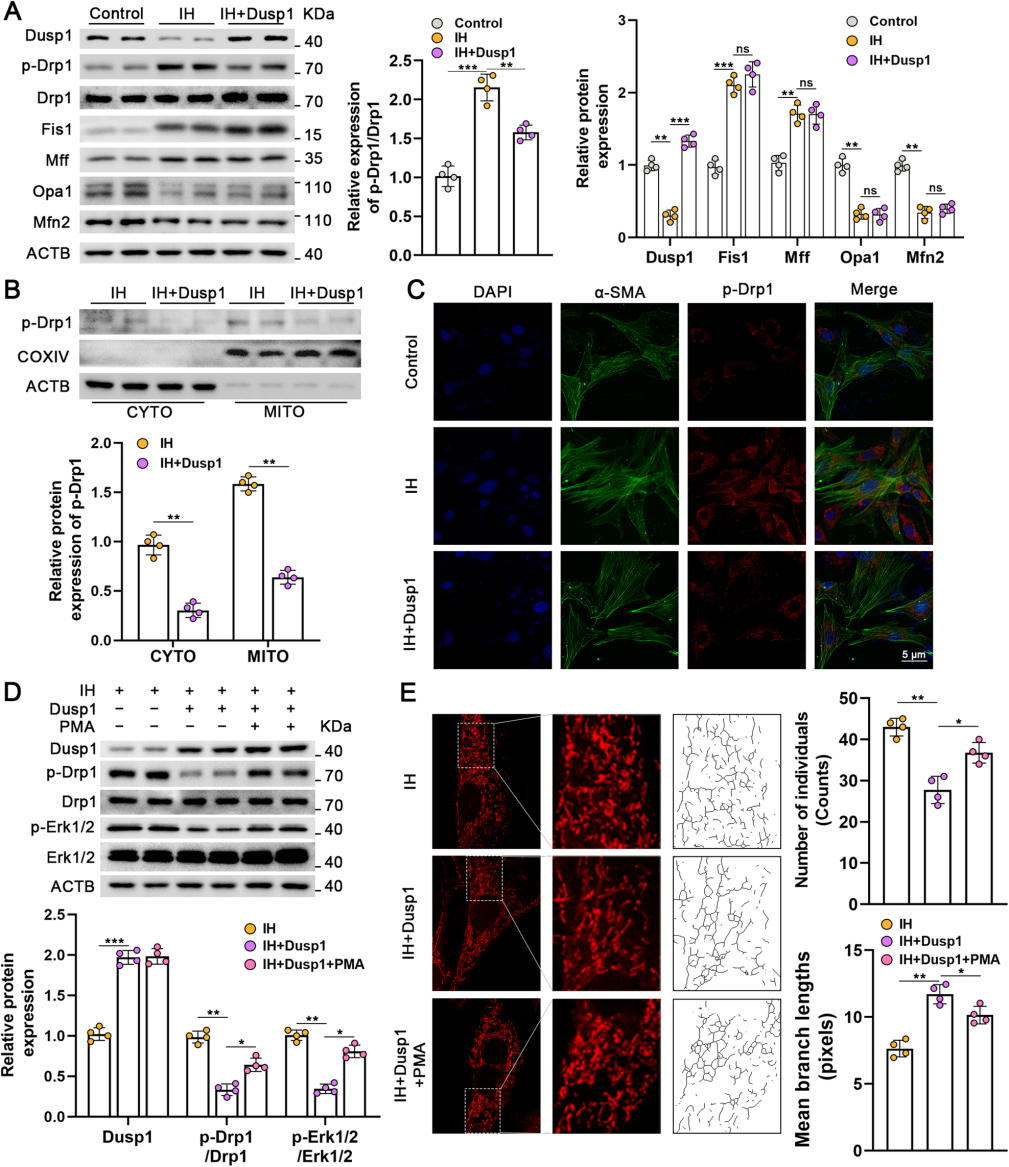
Dusp1 regulates Drp1-mediated mitochondrial fission through the modulation of Erk1/2 phosphorylation. **A** Representative immunoblot images and quantitative analysis of mitochondria fusion and fission-associated proteins following IH or IH+Dusp1 overexpression treatment in PASMCs. **B** Immunoblot analysis and quantification of p-Drp1, Drp1 protein expression following IH, IH+si-Nr1d1, IH+si-Nr1d1+siDusp1 in PASMCs. **C** Cellular immunofluorescence images of p-Drp1 in indicated PASMCs. Alexa Fluor-488 labels α-SMA as green, Alexa Fluor-594 labels p-Drp1 as red, and DAPI counterstains the nuclei in blue, scale bar = 5 µm. **D** Representative immunoblot images and quantitative analysis of Dusp1, Drp1, p-Drp1, p-Erk1/2, Erk1/2 proteins expression in IH-treated PASMCs exposed in Dusp1 or PMA. **E** Confocal images of the mitochondrial network of indicated PASMCs examined by MitoTracker Deep Red, and the number of individual mitochondria as well as the mean mitochondrial branch lengths were calculated, scale bar = 5 µm. Involved 4 biological replicates (*N* = 4). Data are shown as mean ± SD; *P < 0.05 verse IH group; ^#^P < 0.05 verse IH+si-Nr1d1 group.

Initially, we confirmed that intermittent hypoxia leads to Drp1 phosphorylation, an upregulation of Fis1 and Mff, and a downregulation of the fusion proteins opa1 and Mfn2 (Fig. S4A). In contrast, the overexpression of Dusp1 prominently dephosphorylated Drp1 (Fig. 7A, C), while not significantly impacting the expression of other mitochondrial fusion and division proteins (Fis1, Mff, Opa1, Mfn2). Additionally, the Inhibition of Dusp1 counteracted Drp1 dephosphorylation induced by Nr1d1 deficiency. (Fig. 7B). As Dusp1 plays a pivotal role in dephosphorylating the MAPK signaling pathway [18], particularly Erk1/2 signaling involved in regulating Drp1 phosphorylation [19], our conjecture centered on the potential of Dusp1 to modulate Drp1-dependent mitochondrial fission through the regulation of MAPK pathway phosphorylation. The results demonstrated that IH induced phosphorylation of Erk1/2 and Drp1 (Fig. S4A, B). Reversal of these changes was achieved through the use of the Erk1/2 inhibitor U0126, implying the involvement of the Erk1/2 pathway in IH-induced Drp1 phosphorylation (Fig. S4D). In parallel, Dusp1 also elicited inhibition of Erk1/2 and Drp1 phosphorylation (Fig. 7D). Subsequent application of the Erk1/2 pathway activator PMA in these experiments partially restored Erk1/2 and Drp1 phosphorylation, mitigating the effects of Dusp1 overexpression (Fig. 7D). Notably, overexpression of Dusp1 had no significant impact on p-Erk5, p-Juk, and p-p38 in PASMCs (Fig. S4C). Regarding mitochondrial function, we observed that overexpressing Dusp1 also conferred mitochondrial protection, manifesting in the attenuation of IH-induced mitochondrial reactive oxygen species production (Fig. S4E), reduction in membrane potential (Fig. S4F), and mitigation of mitochondrial fragmentation (Fig. 7E). However, it’s noteworthy that these protective effects were reversed by the Erk1/2 agonist PMA. Taken together, these findings suggested that the regulation of Drp1-dependent mitochondrial division by Dusp1 is mediated through the modulation of Erk1/2 phosphorylation.

### Dusp1 knockout exacerbated CIH-induced PH while concurrently enhancing phosphorylation of Erk1/2/Drp1

**Fig. 8:**
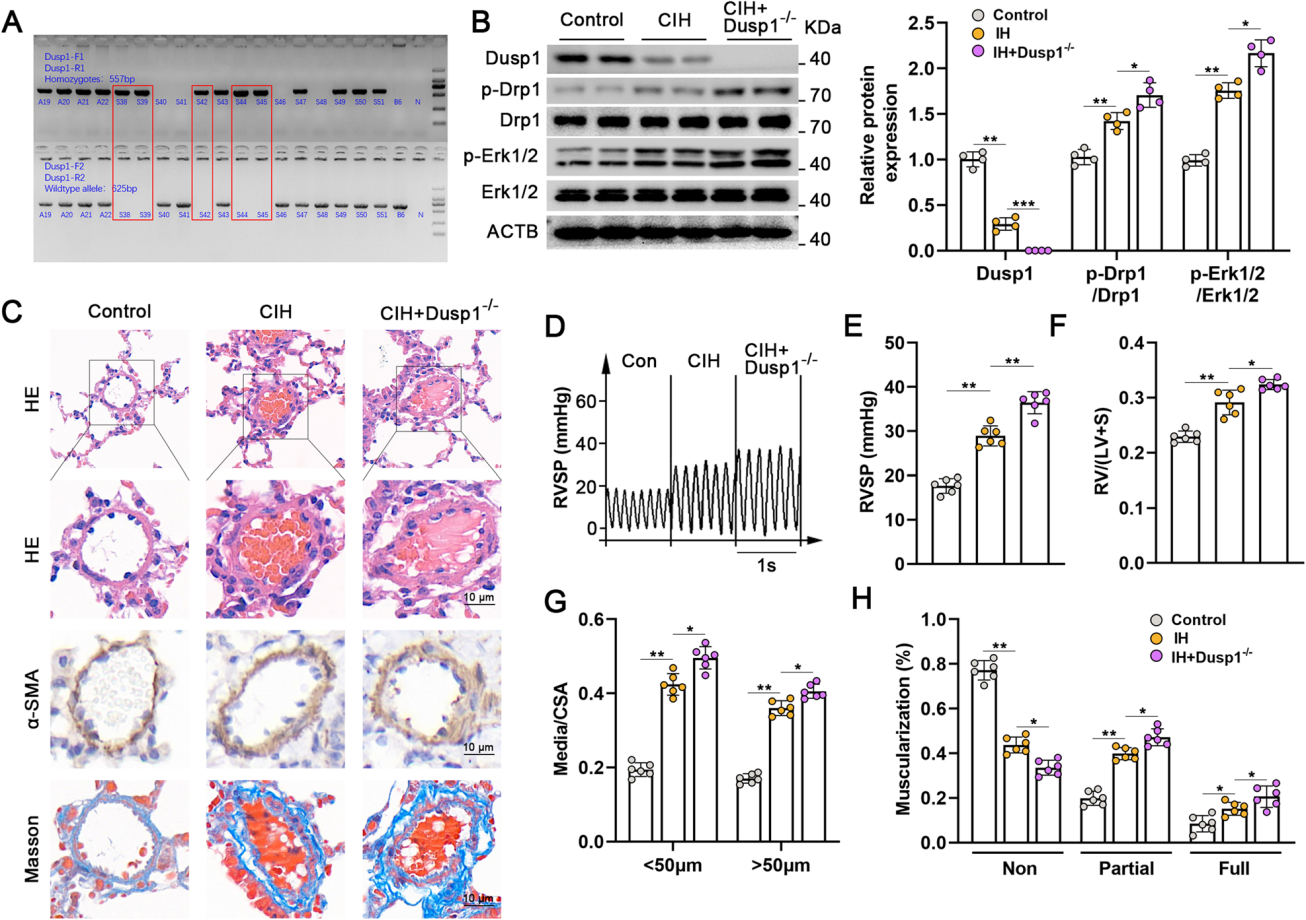
Dusp1 knockdown exacerbates CIH-induced PH by enhancing Erk1/2 and Drp1 phosphorylation. **A** Agarose gel electrophoresis results of Dusp1 knockout mice. B6 is the negative control and N is the blank control. **B** Immunoblot images and quantitative analysis of Dusp1, Drp1, p-Drp1, p-Erk1/2, Erk1/2 proteins expression in mice lung tissue of control group, CIH group, and CIH+Dusp1^−/−^ group. **C** Histological evaluation of mouse lung tissues containing peripheral pulmonary vascular in indicated groups mouse through H&E staining, α-SMA immunohistochemistry, and Masson staining, scale bar = 10 µm. **D** RVSP in three groups of mice. **E** Relative quantitative analysis of RVSP in three groups of mice. **F** Fulton index in indicated groups of mice. **G** Quantification of vascular medial wall thickness in image C. **H** The ratio of non-muscularized, partially muscularized, and fully muscularized pulmonary arterioles in CIH-induced PH mice. Involved 6 biological replicates (*N* = 6). Data are shown as mean ± SD; *P < 0.05 verse Control group; ^#^P < 0.05 verse CIH+ Dusp1^−/−^ group.

Subsequently, we employed Dusp1^−/−^ knockout mice to investigate the in vivo role of Dusp1 in regulating CIH-induced PH. The results of gene identification indicated that lanes S38, S39, S42, S44, and S45 corresponded to Dusp1^−/−^ full knockout mice (Fig. 8A). Compared with CIH group, Dusp1 knockdown significantly enhanced the phosphorylation of Erk1/2 and Drp1 (Fig. 8B), exacerbating remodeling, muscularization of distal pulmonary arteries, and pronounced pulmonary fibrosis around the pulmonary arterial tissues (Fig. 8C, G, H). Furthermore, the CIH+Dusp1^−/−^ group exhibited notably elevated RVSP (Fig. 8D, E) and an increased Fulton index (Fig. 8F), indicating a pronounced development of right ventricular hypertrophy. These findings underscore the significant role of Dusp1 in the development of CIH-induced PH in murine models.

## Discussion

In this study, we have unveiled a previously unrecognized role of the nuclear receptor Nr1d1 in the pathogenesis of IH-induced PH. Firstly, we observed that the expression of the Nr1d1 protein were elevated both in rodent IH-induced PH lung tissues and in human PASMCs. Additionally, our experiments involving AAV1-mediated knockdown of Nr1d1 demonstrated protective effects against PH formation in IH-induced mice and rats PH models. Further mechanistic investigations revealed that Dusp1, a negative regulator of MAPK signaling pathway, served as a direct transcriptional downstream target that Nr1d1 trans-repressed, thus mediating the adverse impacts of Nr1d1 on mitochondrial fragmentation and PH development. Meanwhile, reversal of Dusp1-mediated regulation of Drp1 phosphorylation upon Erk1/2 inhibition with U0123 underscores the pivotal role of the Erk1/2 pathway in governing Drp1 phosphorylation controlled by Dusp1. Finally, intermittent hypoxia-induced a more pronounced development of PH in Dusp1 knockout mice as compared to wild-type mice, primarily through the upregulation of Erk1/2/Drp1 phosphorylation.

Nr1d1 is a nuclear receptor that has garnered significant attention for its pivotal role in governing circadian rhythms and beyond. As a core component of the circadian clock machinery, Nr1d1 operates as a transcriptional repressor, orchestrating the rhythmic expression of numerous target genes involved in diverse physiological processes [20]. Beyond its circadian prowess, Nr1d1 has emerged as a pivotal player in diverse biological arenas. It wields substantial influence over metabolism, directly impacting pathways governing glucose and lipid homeostasis [21]. This metabolic sway has positioned Nr1d1 as a tantalizing therapeutic target for metabolic disorders, including type 2 diabetes and obesity [22]. Additionally, Nr1d1’s regulatory dominion extends to immune responses, as it modulates the inflammatory cascade and influences macrophage function [17]. Furthermore, recent investigations have unveiled its role in controlling autophagy, mitochondrial dynamics, and even neuroprotection [23]. Significantly, emerging evidence underscores the multifaceted influence of Nr1d1 on diverse aspects of physiological and pathological processes encompassing lung inflammation [11], pulmonary fibrosis [24], and cardiovascular dynamics [25]. While prior research has indicated that Nr1d1 activation exerts inhibitory effects on the progression of spontaneous hypertension [26], our investigations have yielded unexpected and profound insights into the role of Nr1d1 in the context of IH-induced PH. Nr1d1 exhibited elevated expression levels in IH-induced rodent PH lung tissues and in human PASMCs. Employing AAV1 to target Nr1d1 in lung tissue, we effectively reduced Nr1d1 expression and mitigated IH-induced PH progression in two distinct animal models. Complementing these in vivo findings, in vitro experiments utilizing PASMCs provided further corroboration of Nr1d1’s deleterious impact. Collectively, these compelling observations furnish indisputable evidence for a hitherto underappreciated role of Nr1d1 as a potent instigator in the pathogenesis of IH-induced PH.

Owing to the absence of activation function 2 (AF2), a motif crucial for co-activator recognition, Nr1d1 is incapable of initiating gene transcription. Instead, its function as transcriptional repressors, harnessing co-repressors like nuclear receptor co-repressor 1 (NCOR1) and histone deacetylase 3 (HDAC3) to curtail gene transcription [17]. In this study, our CHIP-seq and luciferase assay revealed Dusp1 as a direct transcriptional target inhibited by Nr1d1, mediating its adverse effects on mitochondrial fission. Dusp1, also known as mitogen-activated protein kinase phosphatase-1 (MKP-1), regulates a variety of physiological and pathological processes such as inflammation, immune responses, apoptosis, and metabolism by modulating the phosphorylation levels of key molecules in the MAPKs, including Erk1/2, p38, and JNK signaling pathway [18]. One study shown that blood vessel epicardial substance preserves the contractile characteristics of vascular smooth muscle cells through Dusp1-dependent p38MAPK and Erk1/2 signaling, and serves as a safeguard against the development of neointimal [27]. Meanwhile, the Dusp1-Erk1/2 signaling pathway not only controls the homeostatic self-renewal of hepatocytes but also coordinates dynamic shifts in metabolic activity, driven by the period genes [28]. The tight control exerted by Dusp1 on Erk1/2 signaling has profound biological implications. Dysregulation of this balance is implicated in various diseases, including cancer and idiopathic pulmonary fibrosis [29, 30], where overexpression of Dusp1 can lead to decreased Erk1/2 activity and hinder tumor cell and pulmonary fibroblasts proliferation. Conversely, decreased Dusp1 expression can result in excessive Erk1/2 activation, contributing to inflammatory responses and autoimmune diseases [31]. These investigations have underscored the significance of Dusp1 and the Erk1/2 signaling pathway in the preservation of cellular homeostasis.

The multifaceted protein Dusp1 has garnered attention for its significant involvement in maintaining mitochondrial function and mitochondrial fission. Research has shown that promoting Dusp1 expression can restore mitophagy after ischemia in myocardial tissue [32], suppress apoptosis due to neuroinflammation and mitochondrial dysfunction [33], reduce mitochondria-associated oxidative stress damage in sensorineural hearing loss [34], enhance mitochondrial metabolism in obesity [35], and inhibit mitochondrial fragmentation in diabetic nephropathy [36]. In addition, Dusp1 engages in an interaction with VCP, facilitating dephosphorylation and subsequently regulating mitochondrial fusion and fission as a defense mechanism against endotoxemia-triggered myocardial dysfunction [37]. Numerous studies have shown that mitochondrial dysfunction is a hallmark of PH. Impaired mitochondrial respiration, reduced ATP production, increased oxidative stress and excessive mitochondrial fission, all of which contribute to pulmonary vascular remodeling, are commonly observed in PASMCs and pulmonary endothelial cells of PH patient. Epigenetic misregulation of Drp1-interacting proteins MiD49 and MiD51 leads to heightened mitochondrial fission during mitosis, fostering the development of PH [6]. Feng et al. showed that HMGB1 enhanced Drp1 phosphorylation and Drp1-mediated mitochondrial fission via activation of the Erk1/2 signaling, thereby facilitating PASMCs proliferation, migration, and the development of PH [19]. Our consistent findings reveal that Nr1d1-mediated Dusp1 downregulation diminishes mitochondrial respiratory capacity, increases mtROS levels, reduces mitochondrial membrane potential, and amplifies mitochondrial fission. In addition, early Erk1/2 activation orchestrates cellular reprogramming through the promotion of Drp1 phosphorylation and subsequent mitochondrial fission [38]. Furthermore, the influence of Erk1/2 signal on Drp1-depended mitochondrial fission is also evident in sepsis-induced cardiomyopathy and ischemic brain damage [39, 40]. In alignment with these investigations, our study similarly demonstrates that the modulation of mitochondrial fission in IH-induced human PASMCs by Dusp1 is mediated through the Erk1/2-Drp1 signaling.

This study has certain limitations that warrant consideration. Firstly, our focus was primarily on PASMCs, and other crucial cell types such as pulmonary artery endothelial cells and lung fibroblasts were not encompassed in our analysis. Secondly, due to practical constraints, we did not utilize Nr1d1 gene-edited mice, and it’s worth noting that intratracheal injection of adeno-associated viruses could potentially impact bronchial and alveolar epithelial cells. Lastly, to further substantiate the regulatory role of the Nr1d1-Dusp1 axis, it would be essential to incorporate samples from patients suffering from obstructive sleep apnea complicated by pulmonary hypertension.

## Conclusions

In our study, we have substantiated the upregulation of Nr1d1 in IH-induced PH and established the efficacy of Nr1d1 inhibition in ameliorating PH progression. Mechanistically, we have elucidated the role of Nr1d1 in the regulation of the Erk1/2/Drp1 pathway through its interaction with Dusp1, thus influencing mitochondrial function and fission. These discoveries unveil a novel dimension of Nr1d1 involvement in IH-induced PH and bring to light an unexplored axis involving Nr1d1 and Dusp1, which governs mitochondrial fission in PASMCs.

## Materials and methods

### Animals

C57BL/6J mice and 180–240 g male Sprague-Dawley rats, aged 6–8-week-old, were obtained from Hubei Provincial Laboratory Animal Public Service Center (Wuhan, China). Dusp1 knockout (Dusp1-KO) mice were obtained by crossing male and female heterozygous for C57BL/6N-Dusp1e^m1cyagen^ (Presented by Prof. Shi Lang. Strain:19252, Cyagen Biosciences). To ensure statistical significance, each experimental group was randomized to include 6 animals. All animals were kept at a constant temperature of 25 °C in the Animal Laboratory Center, Renmin Hospital of Wuhan University, following a 12-h light/dark cycle. The Renmin Hospital of Wuhan University Institutional Animal Care and Use Committee approved all animal experiments (IACUC Issue No. 20220723A).

### Animal model for IH-induced PH

After 1-week acclimation period, the animals were stratified into normoxia-exposed and chronic intermittent hypoxia (CIH)-exposed using the animal intermittent hypoxia experimental system ProOx-100 (ProOx-100, TOW-INT TECH, China). In the CIH group, rodents were confined within a sealed chamber, where the intermittent hypoxia regimen was precisely controlled with a 120-s cycle. Initially, nitrogen gas was introduced to decrease the inhaled oxygen concentration (FiO_2_) to 6% for 60 s. Subsequently, a rapid infusion of a mixture of air and oxygen was administered to restore FiO_2_ to 21% over 60 s. This cyclic exposure occurred for 8 h daily over a span of 6 weeks for rats and 8 weeks for mice [15, 16]. During all other times, the animals were maintained under normoxic conditions. Animals in the AAV1.Nr1d1 inhibition group underwent intratracheal injections of adeno-associated virus serotype 1 (AAV1) containing Nr1d1 (AAV1.Nr1d1, target sequence for rats: 5′-ACCTATGCCCATGACAAATTA-3′, mouse: 5′-CGTCATAATGAAGCGCTGAAT-3′ and VectorBuilder) after 2 weeks of IH exposure, following induction of anesthesia with isoflurane inhalation. These animals continued to be exposed to IH until the conclusion of the experiment.

### Cell culture and treatment

Human primary PASMCs were procured from iCELL Cellverse Bioscience Technology Co., Ltd. (Shanghai, China, Cat. Number: HUM-iCell-a009,) and cultivated utilizing the Cascade Biologics Medium 231 (M231500, Gibco, USA) containing smooth muscle growth supplement (S00725, Gibco). Transfection of siNr1d1 and siDusp1 (each at 50 nM; si-Nr1d1 target sequence: 5′-CCAGCCCTGAATCCCTCTATA-3′; si-Dusp1 target sequence: 5′- GCACATTCGGGACCAATATAT-3′; Jikai Gene Chemical Co., Ltd, China.) into PASMCs was carried out using Lipofectamine 3000 (Invitrogen, L3000001) in accordance with the manufacturer’s guidelines. Nr1d1 or Dusp1 overexpression plasmids were employed for Nr1d1 or Dusp1 overexpression and constructed utilizing pcDNA3.1. Transfection efficiency was evaluated at 48–72 h post-transfection, and transfected cells were harvested 48 h after transfection for subsequent IH experiments. Concurrently, cellular activity in the Erk1/2 pathway was suppressed using U0126 (HY-12031A, MedChemExpress) with a concentration of 1 μM during a 24-h incubation. Cells in the normoxia group were cultured in a 37 °C incubator with 5% CO_2_ and 21% O_2_. The cellular model for intermittent hypoxia was established in accordance with previously established protocols [41]. All in vitro experiments were conducted with 4 biological replicates.

### Statistical analysis

Statistical analysis employed GraphPad Prism 9.4 (GraphPad Software, CA, USA). Data: mean ± standard deviation (SD). Normality and homoscedasticity were confirmed with Shapiro-Wilk and F-test. Analyzed two-group and multiple-group data with t-tests and one-way ANOVA. Mitochondrial network assessed with Fiji and MiNA macros. P < 0.05 was established for statistical significance. Blinding was not performed in this research. The Supplemental Methods section is available in the Supplemental Material and the antibody information is provided in Table S1.

## Supplementary information

Supplemental Material

Original western blots

## Data Availability

The data used and/or analyzed during the current study are available from the corresponding author upon reasonable request.
