# Supplementary material for: Nr1d1 inhibition mitigates intermittent hypoxia-induced pulmonary hypertension via Dusp1-mediated Erk1/2 deactivation and mitochondrial fission attenuation
Source: Cell Death Discov. 2024 Oct 29;10:459. doi: 10.1038/s41420-024-02219-5 (PMC11522549; doi:10.1038/s41420-024-02219-5)
Supplement: Supplementary file 1 — Supplemental Material [file 41420_2024_2219_MOESM1_ESM.docx]

Supplemental Material

**Nr1d1 Inhibition Mitigates Intermittent Hypoxia-Induced Pulmonary Hypertension via Dusp1-Mediated Erk1/2 Deactivation and Mitochondrial Fission Attenuation**

Zhou Pan^†1^; Yan Yao^†2^; Xu Liu^1^; Yixuan Wang^1^; Xinyue Zhang^1^; Shiqian Zha^1^; Ke Hu^1^*

^1^ Department of Respiratory and Critical Care Medicine, Renmin Hospital of Wuhan University, Wuhan 430060, China.

^2^ Department of Pharmacy, Renmin Hospital of Wuhan University, Wuhan 430060, China.

‬*Corresponding author: Ke Hu, Department of Respiratory and Critical Care Medicine, Renmin Hospital of Wuhan University, Wuhan 430060, China.

E-mail: huke-rmhospital@163.com. ORCID: 0000-0001-9862-7239

^†^ Contributed equally

Supplemental Methods

Supplemental Results

Tables S1

Figures S1-S4

**Supplemental Methods**

**Evaluation of right ventricular function**

Peak right ventricular systolic pressure (RVSP) was assessed as an indicative measure of pulmonary artery systolic pressure, following established procedures [1]. Rodents were anesthetized with isoflurane, and a 25-gauge needle connected to a pressure transducer (YPJ01H, Chengdu Bioinstruments, China) was carefully inserted through the diaphragm into the right ventricle. The lumens were then filled with heparin saline (10 u/ml). RVSP data were meticulously recorded and subjected to analysis using the RM6240 biological signal system (Chengdu Bioinstruments, China). The ratio of the right ventricle to the combined weight of the left ventricle and septum (Fulton Index) was used to determine right ventricular hypertrophy. To evaluate cardiomyocyte cross-sectional area within the right ventricle, FITC-tagged wheat germ agglutinin staining was performed. After the RVSP measurements, euthanasia was performed on all rats by exposing them to a sufficient concentration of CO_2_, followed by the collection of heart and lung tissue samples for subsequent analysis.

**Pulmonary histopathology and immunostaining**

The right lung tissues were promptly excised and flash-frozen in liquid nitrogen to prepare homogenates. Simultaneously, the left lung specimens were immersed in a 4% paraformaldehyde (PFA) solution for a duration of 24 hours. Following this fixation, lung sections were embedded in paraffin, with thicknesses of 5µm, for subsequent hematoxylin-eosin (HE) and α-smooth muscle actin (anti-α-SMA antibody, 1 μg/ml, Abcam, ab7817) staining to facilitate morphological assessments. Our analysis of pulmonary vascular remodeling centered on the evaluation of medial wall thickness, adhering to established protocols [2]. The measurement of medial wall thickness involved the random examination of approximately 20 muscular arteries within the diameter range of 20-50 µm and 50-100 µm in each lung. The determination of medial wall thickness was performed utilizing Image J Fiji (v2.14, https://imagej.net/Fiji), and the calculation was executed using the following formula: wall thickness = (total vascular area - lumen area)/total vascular area. Additionally, the degree of muscularization was ascertained by classifying 40-60 intra-acinar vessels within each mouse as either non-muscular, partially muscular, or fully muscular vessels, in line with previously established criteria [3]. The extent of muscularization was quantified as the ratio of non-muscular, partially muscular, or fully muscular vessels to the total pulmonary vessel count. Furthermore, frozen lung sections (8 μm) were used to observe the localization and expression of the green fluorescent protein carried by AAV1 in lung tissues. For immunofluorescence staining, 5 µm lung tissue paraffin sections or cells on coverslips (fixed with 4% paraformaldehyde) were subjected to a 10-minute permeabilization step with 0.3% Triton X-100. Subsequently, they were blocked with 3% BSA for 1 hour at room temperature. After blocking, samples were incubated overnight at 4°C with primary antibodies: anti-αSMA antibody, (1µg/ml, ab7817, Abcam, USA), anti-Nr1d1 antibody (1:200, 13418, Cell Sigaling, USA), anti-Dusp1 antibody (1:100, sc-373841, Sant Cruz), anti-p-Drp1 antibody (1:500, 4494 Cell Sigaling) followed by washing and incubation with suitable fluorescently labeled secondary antibodies (Alexa Fluor®488 or Alexa Fluor®594, 1:1000, Cell Signaling) for 1 hour at room temperature. Finally, after mounting with an anti-fluorescence quenching medium containing DAPI (G1407-25ml, Servicebio, China), images were captured using a fluorescence microscope (Olympus, Japan), and the analysis was conducted using Image J Fiji.

**Assay for Cellular Proliferation and Migration**

A total of 2×10^4^ normal or transfected PASMCs were seeded into 96-well plates and left to culture overnight. Subsequently, the cells were exposed to either normoxic or IH conditions for 24 hours. Cell viability was assessed by measuring the absorbance at 450nm using the CCK-8 kit (Beyotime, Shanghai, China) according to the provided instructions. Proliferation was assessed using the EdU Cell Proliferation Assay Kit (Beyotime). A wound healing assay was conducted to assess cell proliferation. PASMCs were cultured in 6-well plates with serum-free medium until they achieved 80% confluence. After a scratch was created using a 200-μl plastic pipette tip, the migration distance was measured after 24 hours. For the evaluation of cell migration, a Matrigel chamber (BD Biosciences, USA) was employed following the manufacturer's instructions. Essentially, cells were placed in the upper chamber with serum-free medium, while the lower chamber was filled with 20% fetal bovine serum. After 24 hours, the non-migrated cells on the upper side of the chamber were removed and stained with crystal violet, and they were subsequently photographed and analyzed under a microscope.

**Flow cytometry evaluation of apoptosis**

The PASMCs obtained from each group were subjected to Annexin V-FITC and propidium iodide (PI) staining, following the manufacturer's protocol (BD Biosciences, USA). Cellular apoptosis was then assessed using a flow cytometry detection system.

**Luciferase reporter assay**

Primary human PASMCs were seeded in a 6-well plate and co-transfected with 1 µg of pcDNA3.1-m_Nr1d1 plasmid and pRL-TK control vector (Renilla) (40:1), and 1 µg pGL3-m_wtDusp1 plasmid or pGL3-m_mut Dusp1 plasmid (Firefly). Cell lysates were collected at 48h after transfection using passive cell lysis (R126M; Beyotime, China). Luciferase activities were measured by the Dual Luciferase Reporter Assay System (E1910; Promega, USA) using the SpectraMaxi3 reader.

**ChIP-Seq and ChIP-qPCR analysis**

Chromatin immunoprecipitation assays were performed according to the previously described [4]. Briefly, PASMCs were washed in cold PBS and cross-linked with 1% formaldehyde for 10 minutes at room temperature and then quenched by addition of glycine (125 mmol/L final concentration). Afterwards, samples were lysed with 50mM Tris-HCl (pH 8.0), 10mM EDTA, 1%SDS, 1×protease inhibitor cocktail, and chromatins were obtained on ice. Chromatins were sonicated to get soluble sheared chromatin (average DNA length of 200–500 bp). 20ul chromatin was saved at –20°C for input DNA, and 100ul chromatin was used for immunoprecipitation by anti-Nr1d1 antibodies (13418, Cell Sigaling, USA). 10 μg of antibody was used in the immunoprecipitation reactions at 4 °C overnight. The next day, 30 μL of protein beads was added and the samples were further incubated for 3 h. The beads were next washed once with 20 mM Tris/HCL (pH 8.1), 50 mM NaCl, 2 mM EDTA, 1% Triton X-100, 0.1% SDS; twice with 10 mM Tris/HCL (pH 8.1), 250 mM LiCl, 1 mMEDTA, 1% NP-40, 1% deoxycholic acid; and twice with TE buffer 1× (10 mM Tris-Cl at pH 7.5. 1 mM EDTA). Bound material was then eluted from the beads in 300 μL of elution buffer (100 mM NaHCO3, 1% SDS), treated first with RNase A (final concentration 8 μg/mL) during 6 h at 65°C and then with proteinase K (final concentration 345 μg/mL) overnight at 45°C. Immunoprecipitated DNA was used to construct sequencing libraries following the protocol provided by the I NEXTFLEX® ChIP-Seq Library Prep Kit for Illumina® Sequencing（NOVA-5143-02，Bioo Scientific） and sequenced on Illumina Novaseq 6000 with PE 150 method. DNA samples, including input DNA collected before immunoprecipitation, as well as immunoprecipitated DNA samples, were analyzed using real-time PCR. The subsequent primers were utilized for identifying Nr1d1 binding sites: forward: 5’- TAACTGGGATTACAGGCGCCC-3’; reverse: 5’-CGCTGTGGCTCACACCTGTAA-3’.

**Measurements of mitochondrial superoxide and membrane potential**

Treated PASMCs were incubated in six-well plates and treated under normoxic or IH conditions. The mitochondria-targeting superoxide anion indicator MitoSOX (5 mM, Invitrogen, USA) was used to assay mitochondrial ROS (mtROS) formation. After staining for 15 min, the fluorescence intensity of Mito-SOX was detected by flow cytometry agent. The mitochondrial membrane potential was assessed with the JC-1 kit (Solarbio Life Sciences, Beijing, China) according to standard procedure. Fluorescent images were acquired using a fluorescence microscope (Olympus), and mitochondrial membrane potential depolarization was quantified by calculating the percentage of red/green fluorescence using ImageJ software.

**Mitochondrial oxygen consumption rate (OCR) assay**

Oxygen consumption was measured in a Seahorse XF24 analyzer (Agilent, USA) in accordance with the manufacturer’s instructions. In brief, IH treated PASMCs (2×10^4^ cells/100µl/well) were seeded overnight in an XF24-well culture plate. Wells without cells were used as the background control. Sensor plates were calibrated overnight in a CO_2_-free incubator at 37°C. Prior to the assay, the culture medium was replaced with assay medium [XF DMEM base medium (pH 7.4) with 1 mmol/L pyruvate, 2 mmol/L glutamine, and 10 mmol/L glucose] and the cells were incubated for 1 h in the CO_2_-free incubator at 37°C. Injection ports were loaded with 10× injection mixes to obtain a final concentration of 1.5 µmol/L oligomycin after the first injection, 2.0 µM FCCP after the second injection and 0.5 µM rotenone/antimycin after the third injection using an XF Cell Mito Stress Test Kit (103015–100; Agilent, USA). After the measurement, supernatants were carefully aspirated and protein was extracted. OCR values were normalized to the total protein content of each well.

**Mitochondrial fission analysis**

After labeling the mitochondria with MitoTracker Deep Red (M22426, Invitrogen, USA) according to the supplier's instructions, individual cell images were obtained under a confocal microscope. After processing the acquired images with unsharp mask, local contrast, make binary, and skeletonize, the 'analyze mitochondrial morphology' program of Fiji was employed to calculate the mean branch length and the number of individual mitochondria in each group cells.

**Isolation of PASMCs mitochondrial fractions**

To prepare mitochondrial fractions, we followed the manufacturer's instructions provided with the mitochondrial isolation kit (MdeChemExpress, HY-K1060, New Jersey, USA). Briefly, trypsin buffer was employed for the collection of 2 × 10^7 cultured PASMCs, which were subsequently washed and suspended in 2 ml ice-cold mitochondria isolation reagent (contained 1 mM PMSF) for a duration of 10min. Following homogenization, the cell suspension was subjected to centrifugation at 600g at 4°C for 10 minutes to facilitate the removal of the supernatant, primarily containing cell nuclei and cellular debris. The supernatant was then carefully transferred to a clean centrifuge tube, followed by centrifugation at 11,000g at 4°C for 10 minutes to precipitate the mitochondria. After discarding the supernatant, 150 μl of Mitochondria Lysis Buffer (with 1 mM PMSF) was introduced, and mitochondrial proteins were obtained by centrifugation at 12,000g at 4°C for 10 minutes. Immunoblotting was carried out using 8 μg of mitochondrial protein for each lane.

**Western blotting**

The total protein of lung tissue or PASMCs were extracted with RIPA lysis (contained phosphatase inhibitors A/B, protease inhibitor PMSF, and cocktail, all of which purchased from Servicebio, China). Protein concentration was determined through the BCA method. Next, a denatured protein sample of 30 μg was separated using SDS-PAGE electrophoresis, transferred onto a PVDF membrane (Millipore Corporation, USA). Afterward, the PVDF membrane was blocked with 5% skimmed milk powder for 1 hour at 25°C. Primary antibodies (Table S1) were appropriately diluted and incubated overnight at 4°C. Subsequently, the secondary antibody, goat anti-rabbit (HRP-labeled) IgG (1:5000, Servicebio, China), was applied and allowed to incubate for 1 hours at 25°C. Following ECL chemiluminescence, the blots were quantified using ImageJ software.

**PCR for gene identification**

The genomic DNA extraction from mouse tail samples was carried out using a genomic DNA extraction kit (DP304-02, TIANGEN, China). Subsequently, the DNA concentration and purity were assessed using a Nanodrop ND-1000 (Thermo Fisher Scientific). PCR amplification was conducted following the manufacturer's instructions provided with the Premix TaqTM (RR902Q, TaKaRa, Japan) kit. The amplification conditions and primer sequences were as follows: 95°C for 5min, 95°C for 30s, 58°C for 30s, 72°C for 30s, for a total of 40 cycles; 72°C for 3min; 25°C for hold. Dusp1 Mut primers: forward:5’-TGAGAACGTGATACCAGGAGTG-3’, reverse: 5’- ACCTACACTGGCTTTGTCTGTC -3’; Dusp1 wildtype primers: forward:5’- TGAGAACGTGATACCAGGAGTG -3’, reverse: 5’- GAGCGACAATCCAACAACAGG -3’The PCR products were then subjected to analysis through 2% agarose gel electrophoresis.

**Co-immunoprecipitation (Co-IP)**

The cell lysate was collected, centrifuged, and then incubated with an anti-Nr1d1 primary antibody at 4°C overnight. A lysate immunoprecipitated with anti-IgG (2729; Cell Signaling Technology, USA) was used as a negative control. The immune complexes were subsequently purified using 20 µl of protein G magnetic beads (88847; ThermoFisher, USA) at 4°C for 2 hours, followed by centrifugation and washes with NP40 cell lysis buffer. The immunoprecipitated protein was further analyzed by Western blotting using specific antibodies.

Supplemental Table

Table S1. Antibody used in this study

| Antibody | WB | IHC | IF | Company and catalog |
| --- | --- | --- | --- | --- |
| Nr1d1 | 1:1000 |  | 1:200 | Cell Sigaling, 13418 |
| Bax | 1:1000 |  |  | Cell Signaling, 5023S |
| Bcl2 | 1:1000 |  |  | Abcam, ab182858 |
| Dusp1 | 1:1000 |  | 1:100 | Sant Cruz, sc-373841 |
| α-SMA | 1 µg/ml | 0.05 µg/ml | 1 µg/ml | Abcam, ab7817 |
| PCNA | 1:1000 |  |  | ABclonal, A0264 |
| NcoR1 | 1:1000 |  |  | Cell Signaling, 34271 |
| HDAC3 | 1:1000 |  |  | ABclonal, A19537 |
| P-Drp1 | 1:1000 |  | 1:500 | Cell Signaling, 4494 |
| Drp1 | 1:1000 |  |  | Cell Signaling, 8570 |
| p-Erk1/2 | 1:2000 |  |  | Cell Signaling, 4370 |
| Erk1/2 | 1:2000 |  |  | Cell Signaling, 9102 |
| Fis1 | 1:1000 |  |  | ABclonal, A19666 |
| Mff | 1:1000 |  |  | ABclonal, A8700 |
| Opa1 | 1:2000 |  |  | ABclonal, A9833 |
| Mfn2 | 1:2000 |  |  | Proteintech, 12186-1-AP |
| p-Erk5 | 1:1000 |  |  | Cell Signaling, 3371 |
| p-Jnk | 1:1000 |  |  | Cell Signaling, 4668 |
| p-p38 | 1:1000 |  |  | Cell Signaling,9211 |
| COXIV | 1:5000 |  |  | Proteintech, 11242-1-AP |
| GAPDH | 1:10000 |  |  | Abcam, ab8245 |
| ACTB | 1:5000 |  |  | Abcam, ab8226 |
| IgG | 1:5000 |  |  | Abcam, ab172730 |

**References**

1. J B Snow, C E Norton, M A Sands, L Weise-Cross, S Yan, L M Herbert, et al. Intermittent Hypoxia Augments Pulmonary Vasoconstrictor Reactivity through PKCβ/Mitochondrial Oxidant Signaling. American journal of respiratory cell and molecular biology. 2020;*62*:732-746.

2. A G Hameed, N D Arnold, J Chamberlain, J A Pickworth, C Paiva, S Dawson, et al. Inhibition of tumor necrosis factor-related apoptosis-inducing ligand (TRAIL) reverses experimental pulmonary hypertension. The Journal of experimental medicine. 2012;*209*:1919-1935.

3. R T Schermuly, E Dony, H A Ghofrani, S Pullamsetti, R Savai, M Roth, et al. Reversal of experimental pulmonary hypertension by PDGF inhibition. J Clin Invest. 2005;*115*:2811-2821.

4. S G Landt, G K Marinov, A Kundaje, P Kheradpour, F Pauli, S Batzoglou, et al. ChIP-seq guidelines and practices of the ENCODE and modENCODE consortia. Genome research. 2012;*22*:1813-1831.

**Supplemental Results**


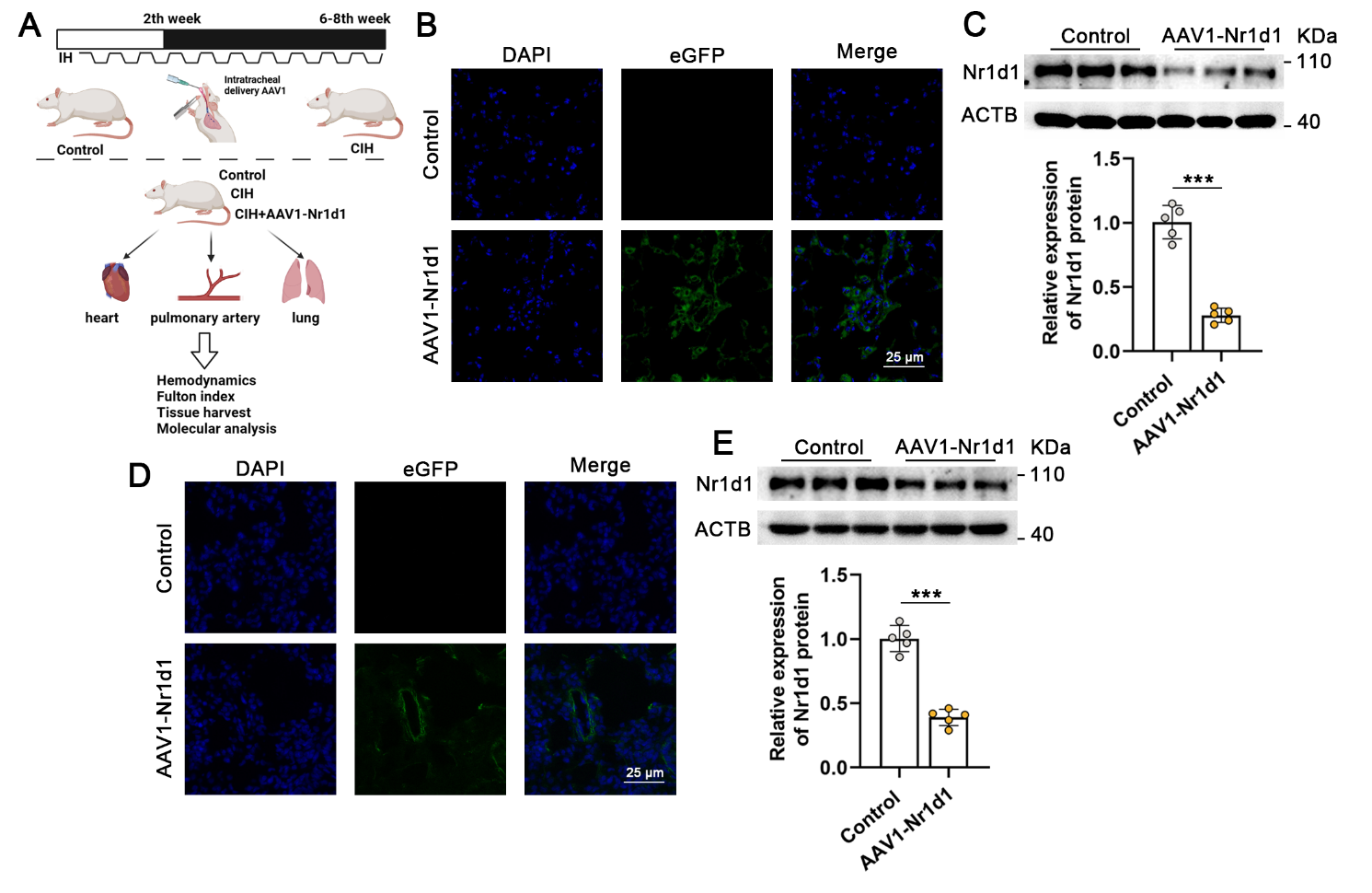


**Figure S1.** (A) Schematic of the experimental design to assess the therapeutic efficacy of AAV1.Nr1d1 gene therapy in the IH-induced PH rat or mouse model. Tissues were collected at week 6 (rats) or 8 (mouse) for molecular and histology analysis. (B-C) The inhibitory effect of AAV1.Nr1d1 detected in a frozen section of rats’ lung tissue and by western blot. (D-E) The inhibitory effect of AAV1.Nr1d1 detected in a frozen section of mouse lung tissue and by western blot. Involved 6 biological replicates (N=6). Data are shown as mean ± SEM; *P < 0.05 verse Control group.


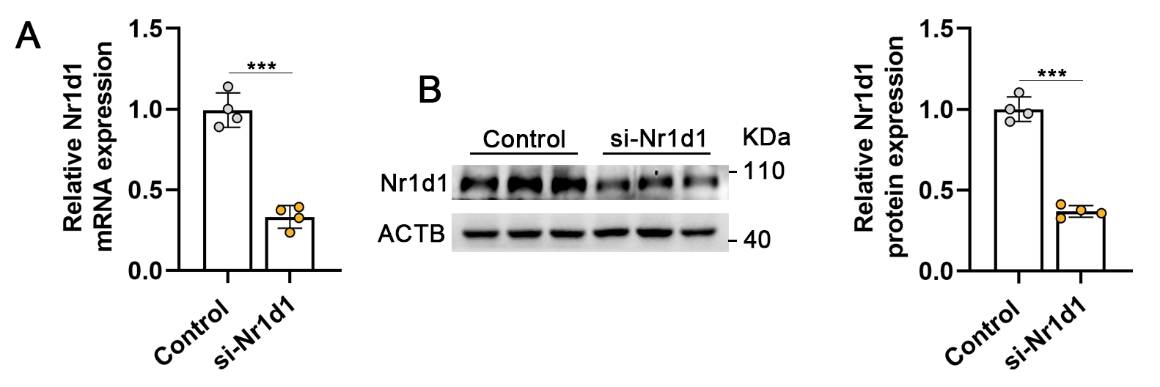


**Figure S2.** (A) mRNA expression of Nr1d1 in PASMCs treated with or without si-Nr1d1. (B) Immunoblotting images and quantitative analysis Nr1d1 protein expression in PASMCs treated with or without si-Nr1d1. Involved 4 biological replicates (N=4). Data are shown as mean ± SEM; *P < 0.05 verse Control group.


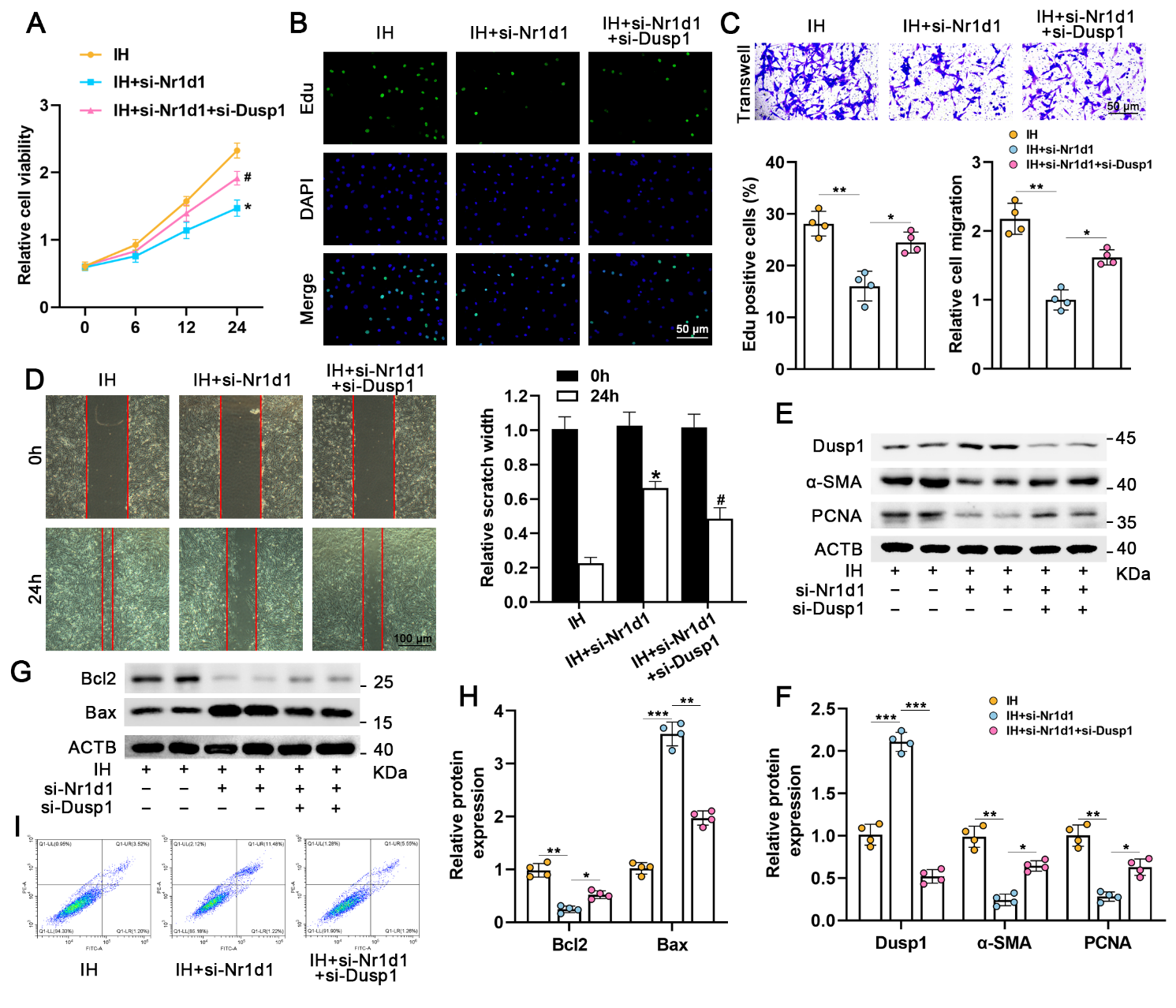


**Figure S3.** (A) Relative cellular activity of PAMSCs in the three groups (IH, IH+si-Nr1d1, IH+si-Nr1d1+si-Dusp1) was assessed at 0, 6, 12, and 24 hours using the CCK-8 kit. (B) Representative images and quantitative analysis of the proliferation in three group PASMCs using the Edu kit; green color represents positive cells, and DAPI stains the nuclei blue, scale bar=50 µm. (C) Representative Tranwell mages and quantitative analysis of the migration in each group of PASMCs. Scale bar=50 µm. (D) Representative Images and quantitative analysis of the migration in each group of PASMCs reflected by wound healing experiments, scale bar=100 µm. (E-F) Representative blots and quantitative analysis of Dusp1, α-SMA and PCNA in the three PASMCs groups. (G-H) Representative blots and quantitative analysis of Bcl2 and Bax in the three PASMCs groups. (I) Flow cytometry of apoptosis in the three PASMCs groups. Involved 4 biological replicates (N=4). Data are shown as mean ± SEM; *P < 0.05 verse IH group; ^#^P < 0.05 verse IH + si-Nr1d1 group.


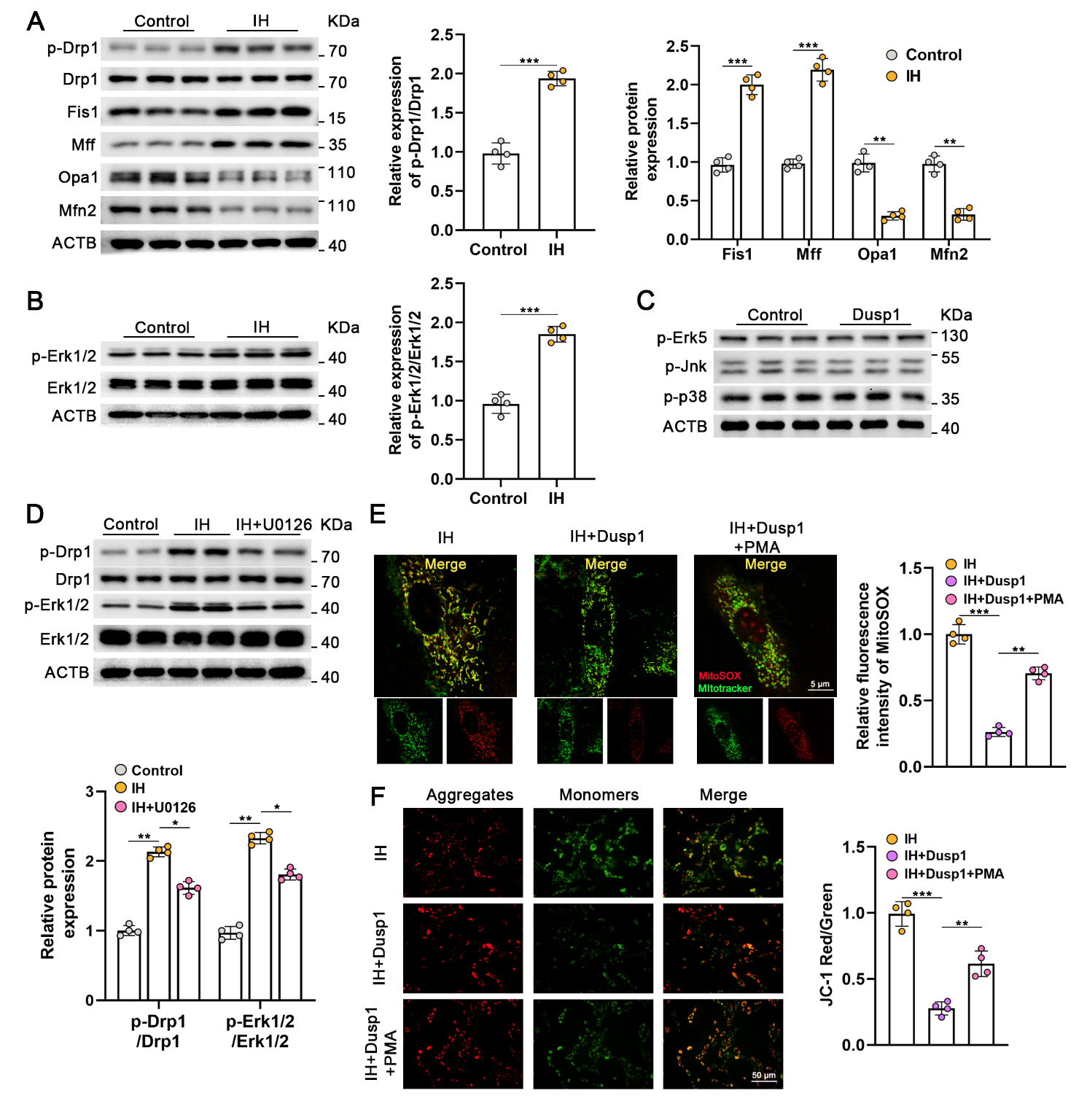


**Figure S4.** (A) Representative immunoblot images and quantitative analysis of mitochondria fusion and fission-associated proteins in PASMCs exposed to normoxic or IH. (B) Immunoblot analysis and quantification of p-Erk1/2 and Erk1/2 in indicated PASMCs. (C) Immunoblots of MAPK signaling pathway-related proteins in PASMCs treated with or without Dusp1 overexpression plasmid. (D) Representative immunoblot images and quantitative analysis of p-Drp1, Drp1, p-Erk1/2, Erk1/2 proteins expression in indicated groups PAMSCs. (E) Representative confocal images and quantification of Mito-SOX (red) and Mito-tracker (green) double staining in three PAMSCs groups, scale bar=5 µm. (F) Image and relative quantitative analysis of mitochondrial membrane potential in various groups of PASMCs using the JC-1 kit, scale bar=50 µm. Involved 4 biological replicates (N=4). Data are shown as mean ± SEM; *P < 0.05 verse Control group; ^#^P < 0.05 verse IH group.
