# Supplementary material for: Nr1d1 inhibition mitigates intermittent hypoxia-induced pulmonary hypertension via Dusp1-mediated Erk1/2 deactivation and mitochondrial fission attenuation
Source: Cell Death Discov. 2024 Oct 29;10:459. doi: 10.1038/s41420-024-02219-5 (PMC11522549; doi:10.1038/s41420-024-02219-5)

Figure 1A

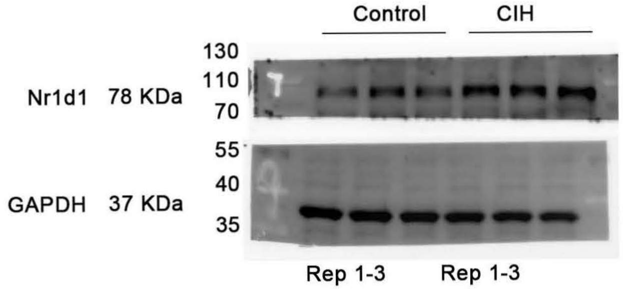

Figure 1C

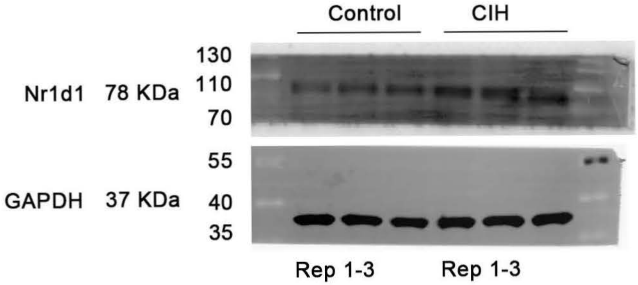

Figure 3E

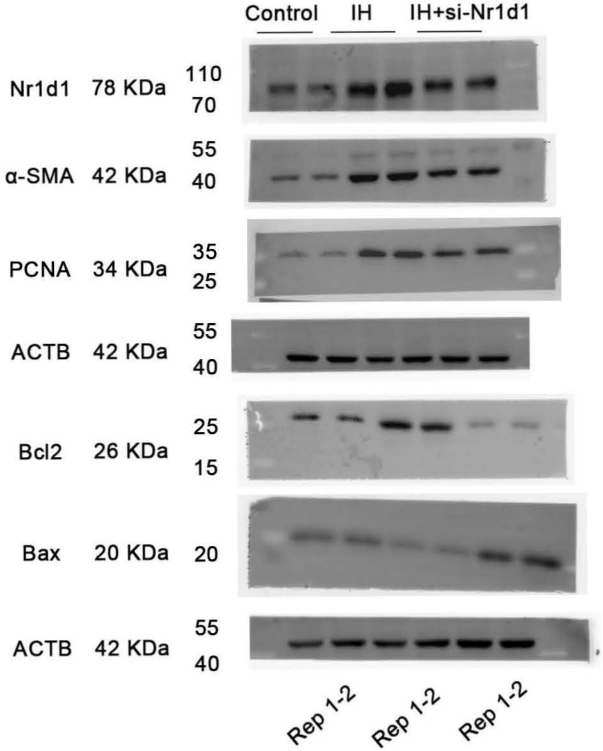

Figure 1E

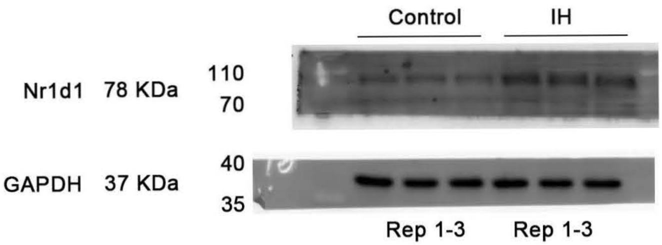

Figure 3E

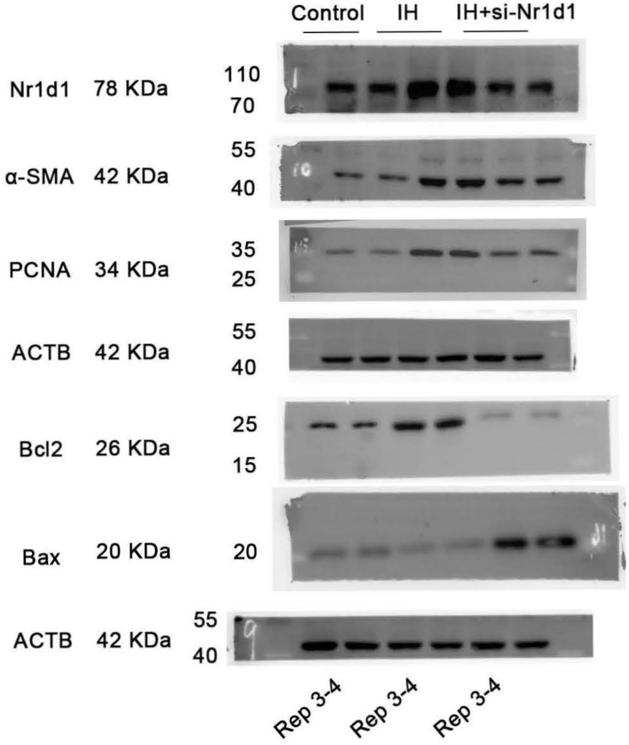

Figure 5H

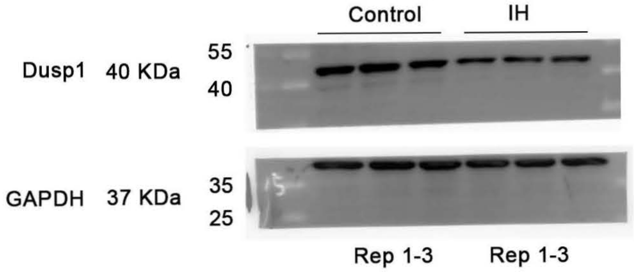

Figure 7A

Figure 7A

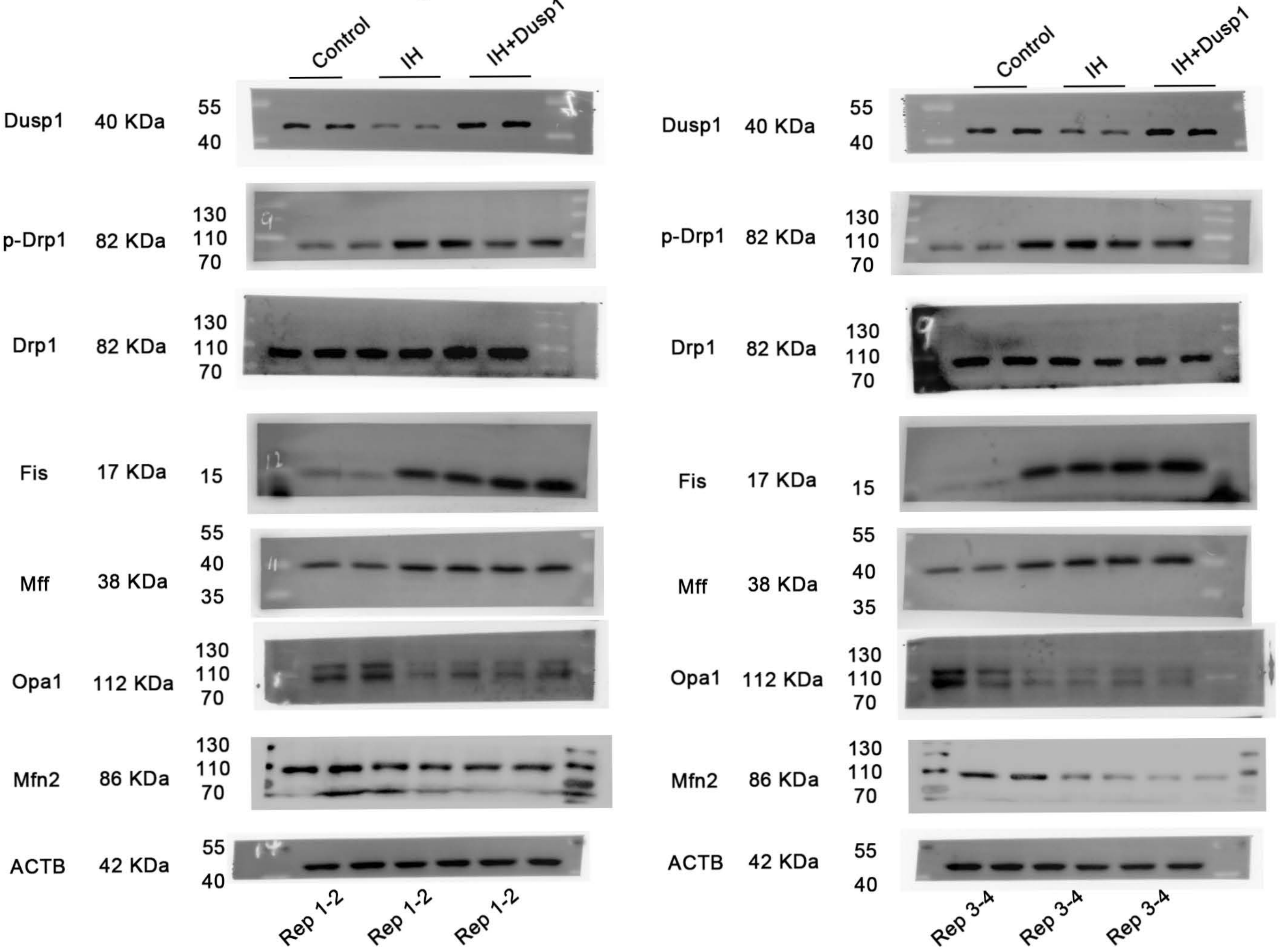

Figure 7B

Figure 7B

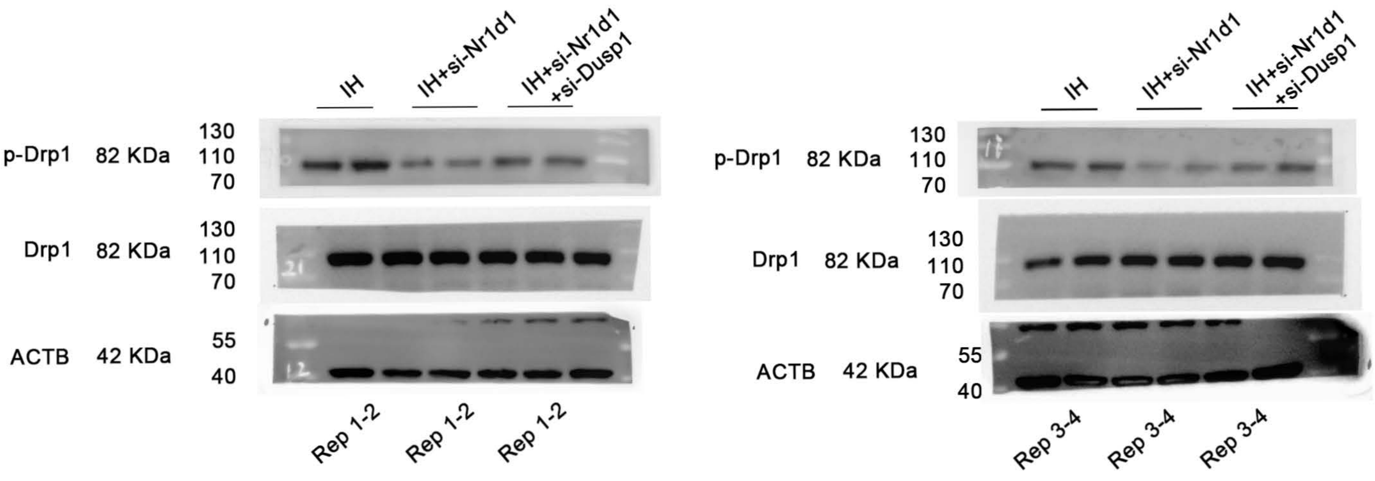

Figure 7D

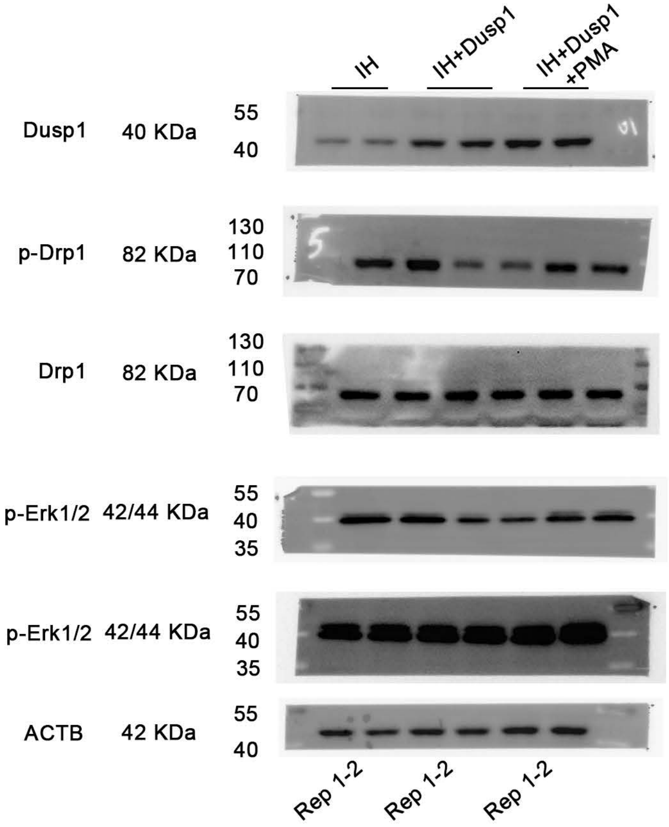

Figure 7D

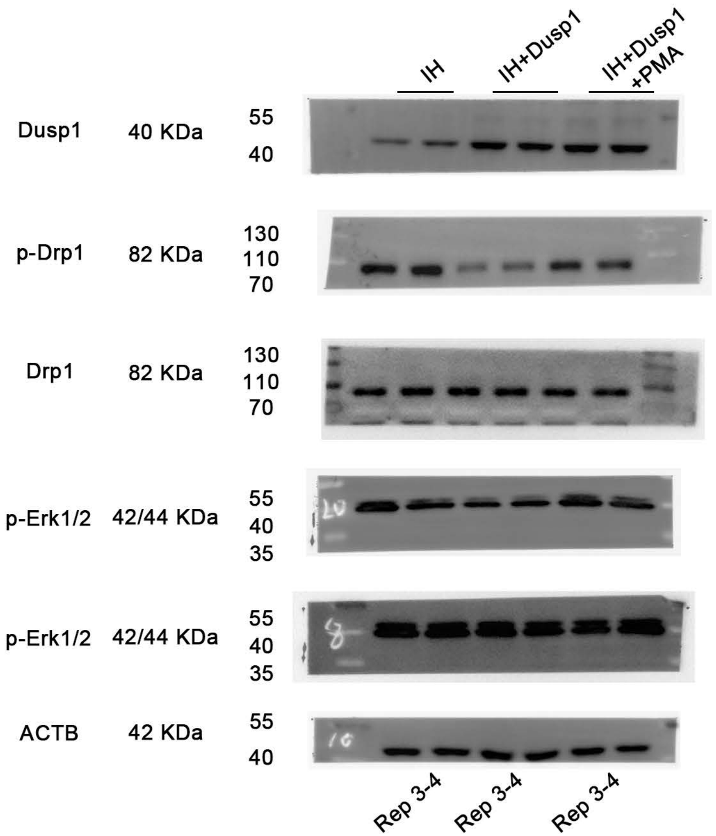

Figure 8B

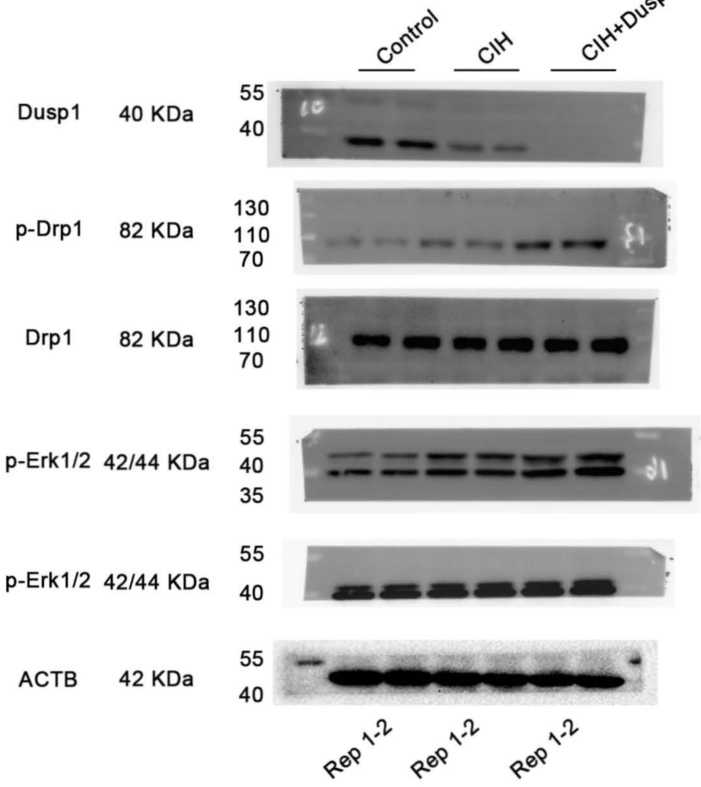

Figure 8B

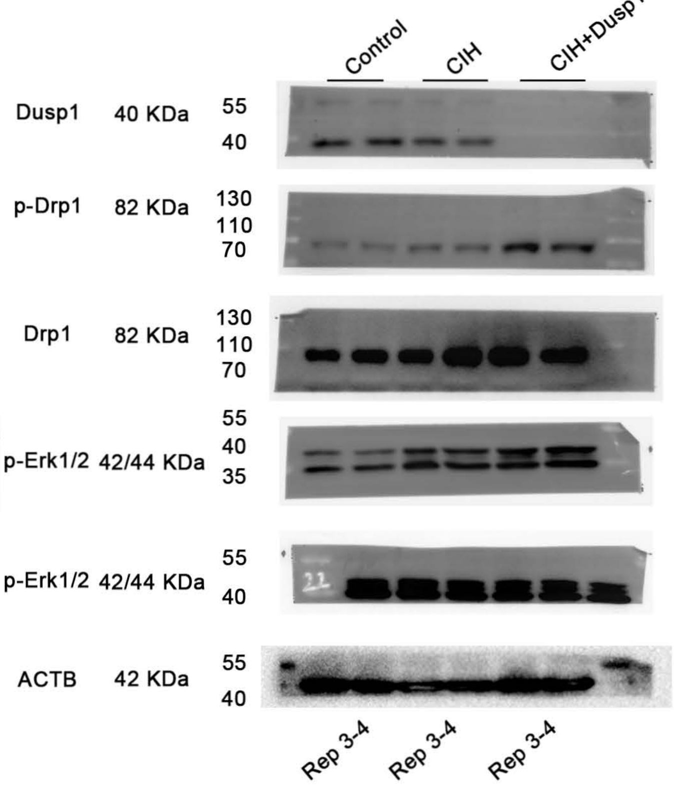

Figure S1C

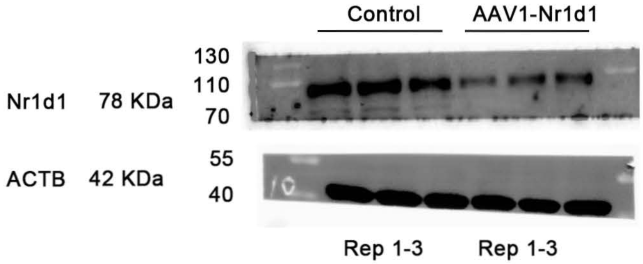

Figure S1E

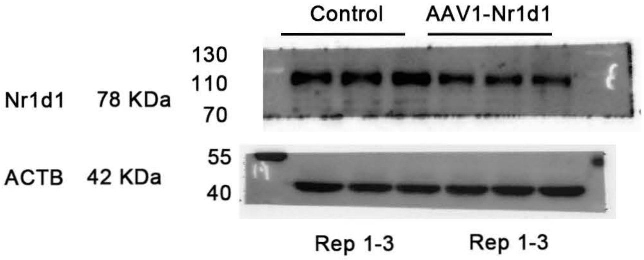

Figure S2B

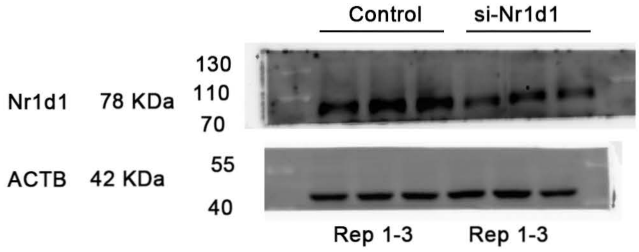

Figure S3E S3G

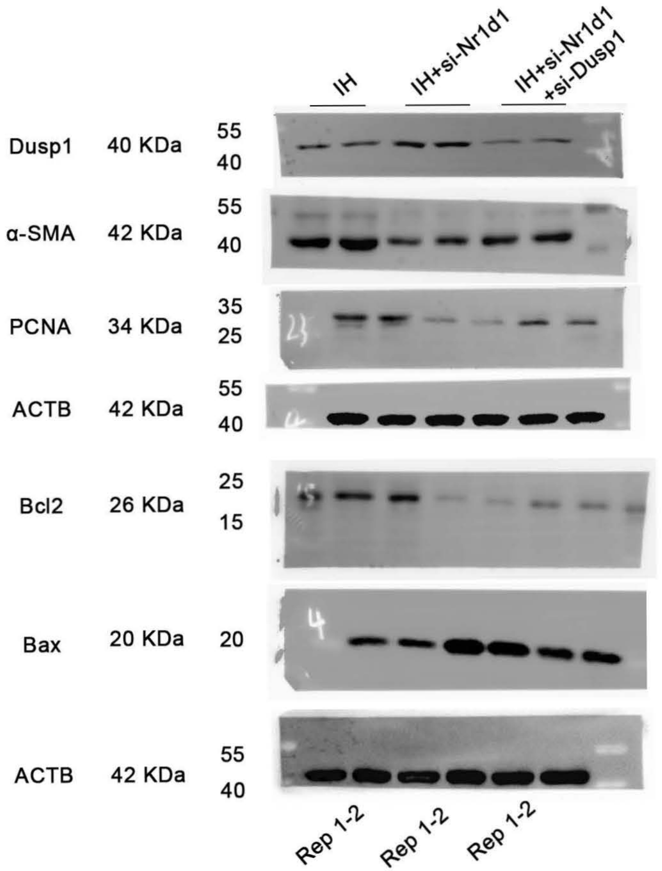

Figure S3E S3G

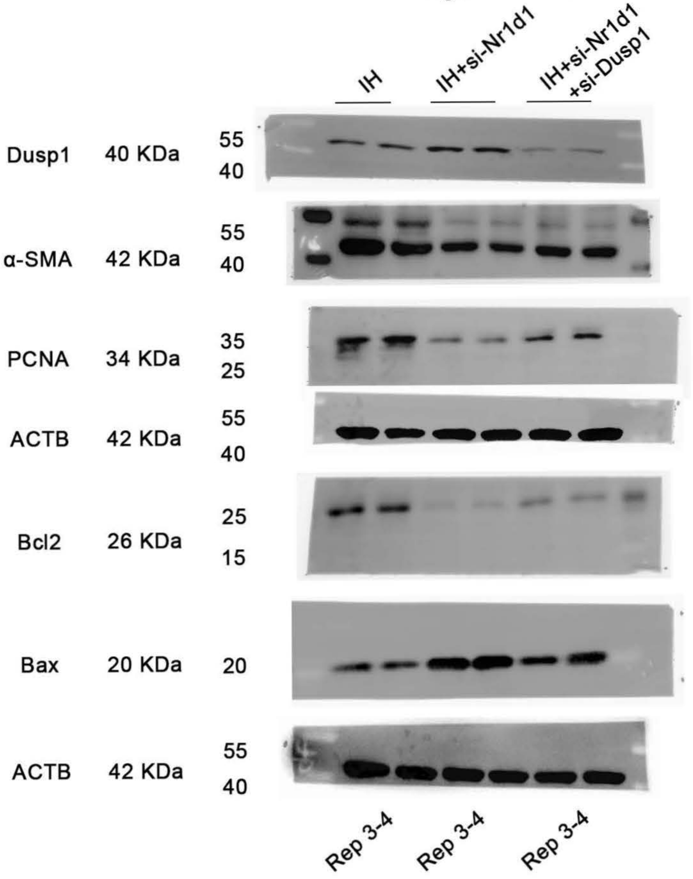

Figure S4A

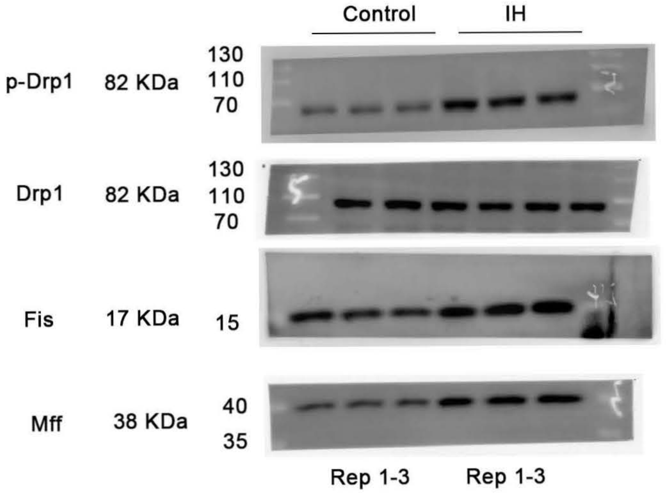

Figure S4A

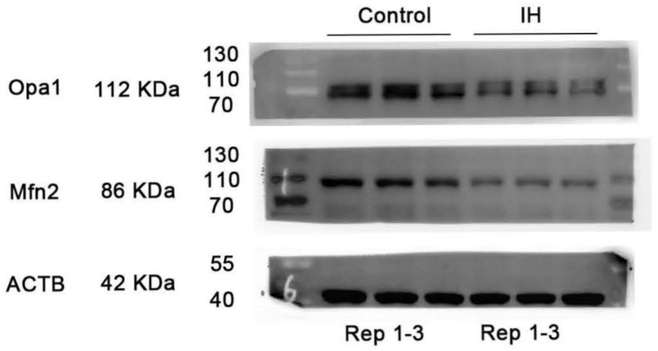

Figure S4B

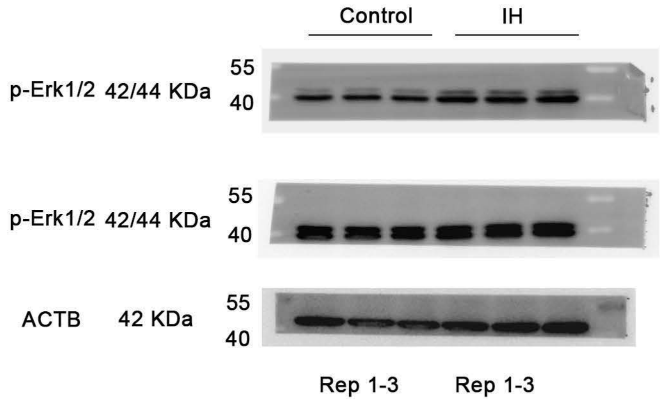

Figure S4C

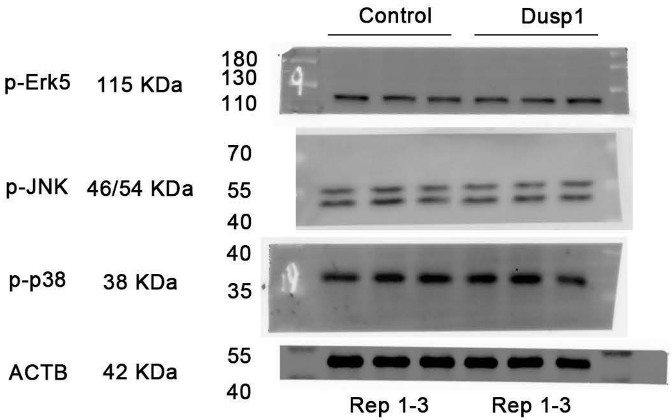

Figure S4D

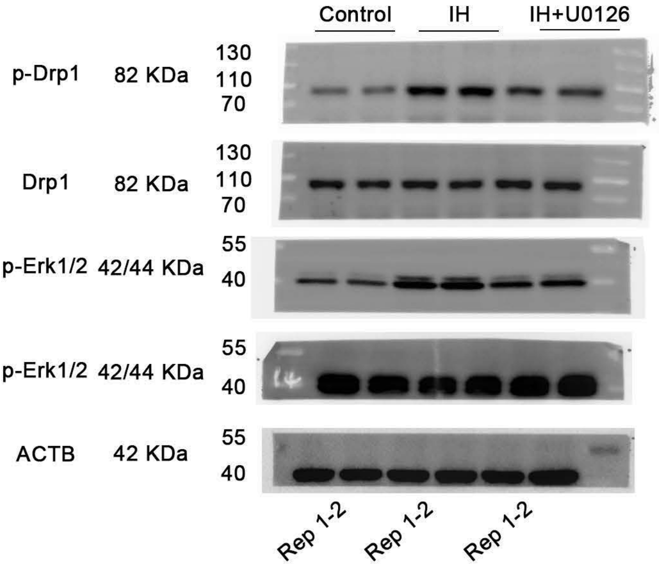

Figure S4D

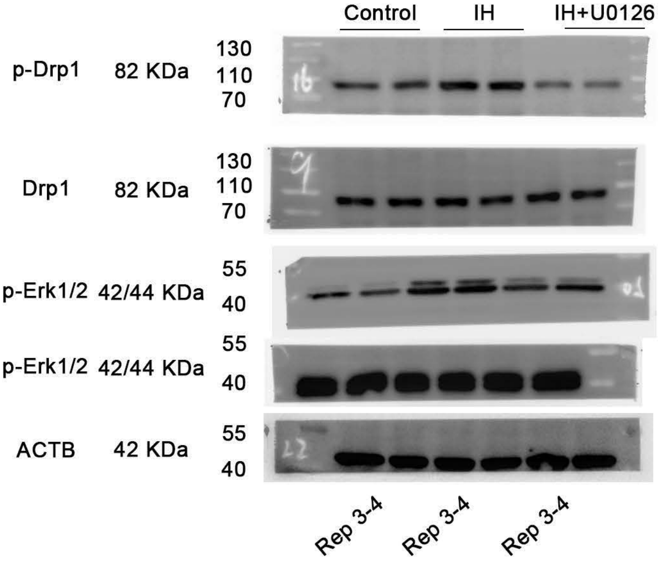

Supplement: Supplementary file 2 — Original western blots [file 41420_2024_2219_MOESM2_ESM.pdf]
